# Key regulator PNPLA8 drives phospholipid reprogramming induced proliferation and migration in triple-negative breast cancer

**DOI:** 10.1186/s13058-023-01742-0

**Published:** 2023-11-28

**Authors:** Zheqiong Tan, Pragney Deme, Keerti Boyapati, Britt S. R. Claes, Annet A. M. Duivenvoorden, Ron M. A. Heeren, Caitlin M. Tressler, Norman James Haughey, Kristine Glunde

**Affiliations:** 1 Russell H. Morgan Department of Radiology and Radiological Science, Division of Cancer Imaging Research, Johns Hopkins University School of Medicine, Baltimore, MD USA; 2 Department of Medical Laboratory, The Central Hospital of Wuhan, Tongji Medical College, Huazhong University of Science and Technology, Wuhan, Hubei China; 3 Department of Neurology, Johns Hopkins University School of Medicine, Baltimore, MD USA; 4 Maastricht MultiModal Molecular Imaging Institute, Maastricht University, Maastricht, The Netherlands; 5 Department of Surgery, NUTRIM School of Nutrition and Translational Research in Metabolism, Maastricht University, Maastricht, The Netherlands; 6 Department of Psychiatry, Johns Hopkins University School of Medicine, Baltimore, MD USA; 7 Sidney Kimmel Comprehensive Cancer Center, Johns Hopkins University School of Medicine, Baltimore, MD USA; 8 Department of Biological Chemistry, Johns Hopkins University School of Medicine, Baltimore, MD USA

**Keywords:** PNPLA8, Breast cancer, Triple negative, Phospholipid, Metabolism reprogramming, Eicosanoids, Migration, Invasion, Proliferation

## Abstract

**Background:**

Triple-negative breast cancer (TNBC) is the most aggressive breast cancer subtype and leads to the poorest patient outcomes despite surgery and chemotherapy treatment. Exploring new molecular mechanisms of TNBC that could lead to the development of novel molecular targets are critically important for improving therapeutic options for treating TNBC.

**Methods:**

We sought to identify novel therapeutic targets in TNBC by combining genomic and functional studies with lipidomic analysis, which included mechanistic studies to elucidate the pathways that tie lipid profile to critical cancer cell properties. Our studies were performed in a large panel of human breast cancer cell lines and patient samples.

**Results:**

Comprehensive lipid profiling revealed that phospholipid metabolism is reprogrammed in TNBC cells. We discovered that patatin-like phospholipase domain-containing lipase 8 (PNPLA8) is overexpressed in TNBC cell lines and tissues from breast cancer patients. Silencing of PNPLA8 disrupted phospholipid metabolic reprogramming in TNBC, particularly affecting the levels of phosphatidylglycerol (PG), phosphatidylcholine (PC), lysophosphatidylcholine (LPC) and glycerophosphocholine (GPC). We showed that PNPLA8 is essential in regulating cell viability, migration and antioxidation in TNBC cells and promoted arachidonic acid and eicosanoid production, which in turn activated PI3K/Akt/Gsk3β and MAPK signaling.

**Conclusions:**

Our study highlights PNPLA8 as key regulator of phospholipid metabolic reprogramming and malignant phenotypes in TNBC, which could be further developed as a novel molecular treatment target.

**Supplementary Information:**

The online version contains supplementary material available at 10.1186/s13058-023-01742-0.

## Introduction

Breast cancer has now surpassed lung cancer as the most commonly diagnosed cancer worldwide and is the leading cancer-related cause of death in women [1]. Breast cancer is a heterogeneous disease and can be categorized into four molecular subtypes according to the definition of the 2013 St. Gallen International Breast Cancer Conference: luminal A, luminal B, human epidermal growth factor receptor 2 (HER2)-positive and triple-negative breast cancer (TNBC) [2]. TNBC is characterized by lack of expression of estrogen receptor (ER), progesterone receptor (PR) and HER2. TNBC accounts for nearly 15–20% of all breast cancers. It is the most aggressive breast cancer subtype and leads to the poorest patient outcomes [3]. The claudin-low breast cancer subtype of TNBC has low expression levels of cell–cell adhesion molecules and high expression levels of epithelial-mesenchymal transition (EMT) and stem cell-like markers, making it the most highly metastatic and most aggressive subtype [4, 5]. The mortality rate of TNBC patients is approximately 42.2% within the first five years after diagnosis, which is significantly higher than the mortality rate (28.8%) of breast cancer patients with other subtypes [6]. Due to its molecular phenotype, TNBC is not sensitive to endocrine and traditional targeted therapies. Therefore, neoadjuvant chemotherapy has become the main treatment approach for TNBC patients [7]. However, recurrence and metastatic rates remain high in TNBC patients because of chemotherapy resistance [8]. Claudin-low TNBC tumors are even less sensitive to chemotherapy than other TNBC tumors [9]. Therefore, exploring new molecular mechanisms of TNBC, especially of the claudin-low TNBC subtype, which could lead to the development of novel molecular targets, are critically important for improving therapeutic options for treating TNBC.

Metabolic reprogramming is a hallmark of cancer and has provided promising therapeutic targets for the treatment of breast cancer [10]. Phospholipid metabolism plays crucial and diverse biological roles in cancer cells, including in energy storage, signal transduction, vesicular trafficking, apoptosis, cell adhesion, and migration [11]. Phospholipids (PLs) are the main building block of cell membranes and act as lipid second messengers for oncogenic signal transduction in breast cancer [12]. The major phospholipids in mammalian cells include phosphatidylcholine (PC), phosphatidylethanolamine (PE), phosphatidylserine (PS), phosphatidylinositol (PI), phosphatidylglycerol (PG), phosphatidic acid (PA), cardiolipin (CL) and sphingomyelin (SM) [13]. PC and PE are the most and the second most abundant phospholipids in mammalian membranes [14]. Alterations in phospholipid metabolism are associated with cancer and other diseases including atherosclerosis and nonalcoholic fatty liver disease [15].

Liquid chromatography-tandem mass spectrometry (LC–MS/MS)-based lipidomic analysis techniques have become useful approaches in cancer research as they reveal the global composition of a large number of lipid species [16]. Lipidomic approaches in cancer research have created new opportunities for understanding the functions and mechanisms of lipids in cancer cells. Lipidomic analysis of phospholipids in human mammary epithelial cells *versus* breast cancer cells has previously indicated that an increase in cellular PC O-16:0/18:1, PC O-16:0/20:1 and PI 22:5/18:0 levels was associated with metastatic potential in breast cancer cells [17]. Several PGs, reported as anti-inflammatory lipids, were decreased in breast cancer cells, compared with nonmalignant breast cells [18]. Significantly higher SM levels and lower lysophosphatidylcholine (LPC) levels were observed in the plasma of breast cancer patients compared with healthy controls [19]. Moreover, LPC 16:0, PC 34:2, PC 42:5 and SM 20:2 were identified as biomarkers for differentiating breast cancer patients from healthy controls [19]. A matrix-assisted laser desorption/ionization-mass spectrometry (MALDI-MS) study indicated that PC 30:0, PC 32:0, PC 32:2, PE 34:1 and PE 35:0 were able to distinguish breast cancer tissues from normal breast tissues [19]. These studies provided solid evidence that phospholipid reprogramming is associated with breast cancer progression. However, developing reliable lipidomic approaches for the diagnosis and treatment of breast cancer will require a deeper mechanistic understanding of key enzymes involved in phospholipid metabolism.

Phospholipases are a family of enzymes that hydrolyze phospholipid substrates. The major classes of phospholipases include phospholipase A1 (PLA1), phospholipase A2 (PLA2), phospholipase B (PLB), phospholipase C (PLC) and phospholipase D (PLD) which catalyze hydrolysis at different positions in phospholipid molecules [20]. PLA1 and PLA2 enzymes hydrolyze sn-1 and sn-2 positions of glycerophospholipids to produce free fatty acids and lysophospholipids. Some PLA2 enzymes both have phospholipase and lysophospholipase activity, which could remove two fatty acids from glycerophospholipids to produce glycerophosphocholine (GPC). PLA2 is a primary regulator of the arachidonic acid cascade and activates a variety of signaling pathways through lipid mediators [21]. PLA2 is generally divided into six families: cytosolic PLA2 (cPLA2), calcium-independent PLA2 (iPLA2), secreted PLA2 (sPLA2), lysosomal PLA2, platelet-activating factor (PAF), and adipose-specific PLA2 [20]. cPLA2α (PLA2G4A), iPLA2β (PLA2G6), sPLA2-IIA (PLA2G2A) and sPLA2-III (PLA2G3) are recognized to play a tumorigenic role in cancer [20]. However, the other PLA2s are not well-studied in cancer.

In this study, we sought to identify novel therapeutic targets in TNBC by combining genomic and functional studies with lipidomic analysis, which included mechanistic studies to elucidate the pathways that tie these lipid biomarkers to critical cancer cell properties. Our study revealed that phospholipid metabolism is reprogrammed in TNBC cells. We showed that PNPLA8 is overexpressed in breast cancer cells and tissues, especially in TNBC cells and tissues, and is essential in regulating cell viability, migration and antioxidation in TNBC cells. Silencing of PNPLA8 disrupts phospholipid metabolic reprogramming in TNBC, particularly affecting PC, PG and GPC levels. Moreover, silencing of PNPLA8 inhibits the arachidonic acid cascade and eicosanoid production in TNBC, which may affect critical cancer cell behaviors through the PI3K/Akt/GSK3β and Mek/Erk pathways. Our study suggests that PNPLA8 is a key regulator of phospholipid metabolic reprogramming and malignant phenotype in TNBC, which opens up new horizons of intrasurgical margin detection, diagnosis, and a new treatment target.

## Methods

### Cell lines and culture conditions

The human non-tumorigenic epithelial cell lines MCF10A and MCF12A were cultured in DMEM/F12 medium (Thermo Fisher Scientific, Waltham, US) with 5% Horse Serum (Thermo Fisher Scientific), 20 ng/mL EGF (Sigma-Aldrich, St. Louis, US), 10 μg/mL insulin (Sigma-Aldrich), 100 ng/mL cholera toxin (Sigma-Aldrich) and 0.5 mg/mL hydrocortisone (Sigma-Aldrich). The human breast cancer cell line MCF7 (RRID: CVCL_0031) was cultured in MEM medium (Thermo Fisher Scientific) with 10% fetal bovine serum (FBS, Thermo Fisher Scientific). The human breast cancer cell lines T47D (RRID: CVCL_0553) and BT474 (RRID: CVCL_0179) were cultured in RPMI medium (Thermo Fisher Scientific) with 10% FBS. The human breast cancer cell line SKBR3 (RRID: CVCL_0033) was cultured in McCoys 5A medium (Thermo Fisher Scientific) with 10% FBS. The human breast cancer cell lines MDA-MB-231 (RRID: CVCL_0062), MDA-MB-468 (RRID: CVCL_0419) and Hs578T (RRID: CVCL_0332) were cultured in DMEM (Thermo Fisher Scientific) with 10% FBS. All cell lines listed above were purchased from the American Type Culture Collection (ATCC, Manassas, US). Human breast cancer cell lines SUM149PT (RRID: CVCL_3422) and SUM159PT (RRID: CVCL_5423) were purchased from BioIVT (Detroit, MI, US) and cultured in Ham’s F12 medium (Thermo Fisher Scientific) with 10% FBS. Cells were incubated at 37 °C with 5% CO_2_ and a humidified atmosphere. All cell lines were annually tested to be free from mycoplasma, and STR profiling was performed to verify cell line identity.

### siRNA silencing

On-target plus Human PNPLA8 siRNA-SMARTpool and negative scrambled siRNA were purchased from Dharmacon (Cambridge, UK). The target sequences of PNPLA8 are listed below: GAGAAGGGCUGUUGCUAAU, UCAGUAACUUGAUGGAUUU, GACCUGAAACAUCGAUUUA and GAGUCUCAUUUGUCCAAUA.

### LC–MS/MS lipidomic analysis

Cell lines were cultured under standard condition as described above for 48 h. Approximately 1 × 10^7^ cells growing in the log phase with a confluence of 80% were trypsinized and pelleted. 200 µL ddH_2_O were added to pellets, followed by sonication with short bursts for homogenization. 5 µL of homogenate was used for BCA protein assay for normalization, and the remaining sample was extracted using a modified Bligh and Dyer procedure to obtain a crude lipid fraction [22]. Cell homogenate samples were gently mixed in a glass tube with ddH_2_O, followed by extraction with methanol/dichloromethane containing twelve internal standards. Following incubation on ice for 30 min and centrifugation (10 min, 3000 g, 4 °C) for phase separation, the organic phase containing lipids was collected and stored at -20 °C. Prior to analysis, 800 μL aliquot of the organic layer was dried and re-suspended in 150 µl of running solvent (dichloromethane:methanol (1:1) containing 5 mM ammonium acetate). Lipid analysis was conducted in MS/MS^ALL^ electrospray ion positive mode on a TripleTOF 5600 (AB Sciex, Redwood City, CA) quadrupole-time of-flight mass spectrometer (Q-TOF) coupled to a high-performance liquid chromatograph (Shimazu, Canby, OR) using a LC-20AD pump and SIL-20AC XR autosampler. The mass spectrometer was operated at a mass resolution of 30,000 for TOF MS scan and 15,000 for product ion scan (MS/MS) in high sensitivity mode, and the instrument was automatically calibrated after every ten-sample injection using an APCI positive calibration solution delivered through an automatic calibration delivery system (AB SCIEX). Details of the mass spectrometry method were previously published [23] and are detailed in the Supplemental Information. MultiQuant software and LipidView database (version 1.3, AB SCIEX, Concord, Ontario, Canada) were used for identification and annotation of lipid species. For relative quantification, lipid peak intensities were normalized using their corresponding internal standards. Each sample was run in duplicate and averaged normalized intensities of each lipid were used for statistical analysis. Detailed Triple TOF MS/MS^ALL^ comprehensive lipidomic analysis protocols are described in supplemental methods. All chemicals and solvents used in this study are summarized in Additional file 1: Table S1.

### Dual-phase extraction and ^1^H magnetic resonance spectroscopy

Cell pellets (at least 1 × 10^7^ cells) were harvested and ground over liquid nitrogen. Water-soluble metabolites of cells were extracted using the dual-phase extraction method (methanol:chloroform:water = 1:1:1) as previously described [24]. The aqueous fractions were lyophilized and re-dissolved in D_2_O containing 0.24 × 10 ^−6^ mol 3-(trimethylsilyl)propionic-2,2,3,3,-d4 acid (TSP, Sigma-Aldrich) as chemical shift and concentration reference for metabolite quantification. Fully relaxed ^1^H high-resolution (HR) magnetic resonance spectroscopy (MRS) was performed using a Bruker Avance-III 750 MHz spectrometer equipped with a 5-mm TXI probe. Water-suppressed spectra were acquired using a 1D NOESY pulse sequence with a relaxation delay of 10 s, 256 scans, 8 dummy scans, receiver gain 40.3, and mixing-time of 80 ms. Water-soluble metabolites were quantified using TopSpin software (Bruker BioSpin Corp., Billerica, MA) as previously described [25].

### RNA extraction and quantitative RT-PCR

RNA extractions were performed using the RNeasy Mini Kit (QIAGEN, Hilden, Germany) following the manufacturer’s protocol. 500 ng of RNA was reversely transcribed using iScript cDNA Synthesis Kit (Bio-Rad, Hercules, US). Quantitative PCR (qPCR) analysis was performed with the CFX Connect Real-Time PCR System (Bio-Rad) using the IQ SYBR Green Supermix (Bio-Rad). The housekeeping gene actin beta (ACTB) was used as an internal control. The relative fold changes in gene expression were calculated using the 2^−△△Ct^ method. Primer sequences are provided in Additional file 1: Table S2.

### Western blots

Cell pellets (at least 1 × 10^6^ cells) were harvested and suspended in RIPA lysis buffer (Sigma-Aldrich) with Protease and Phosphatase Inhibitor Cocktail (Thermo Fisher Scientific). Samples were homogenized by sonication with short burst and centrifuged at 13,000 rpm for 20 min at 4℃. Supernatants were collected, and protein concentration was measured using a BCA Protein Assay Kit (Thermo Fisher Scientific). Proteins (30 μg) were analyzed by SDS-PAGE and transferred onto PVDF membranes (EMD Millipore, Darmstadt, Germany). Following blocking with 5% non-fat milk (EMD Millipore) in Phosphate Buffered Saline (PBS) at room temperature for 1 h, membranes were incubated with primary antibodies overnight at 4℃. Then membranes were incubated with horseradish peroxidase-conjugated secondary antibodies at room temperature for 1 h. Visualization was performed using the Pierce™ ECL Plus Western Blotting Substrate (Thermo Fisher Scientific) and ChemiDoc MP Imaging System (Bio-Rad). The density of the bands was analyzed by Image Laboratory software version 4.0.1 (Bio-Rad). GAPDH was used for normalization. Full uncropped images are shown in Additional files 1: Fig. S4–7. Antibody information is provided in supplemental materials. All antibodies used in this study are summarized in Additional file 1: Table S1.

### Immunohistochemistry of TMAs

Two human breast cancer tissue microarray (TMA) slides (catalogue #BR1921C; Biomax, Rockville, MD and catalog #BC081120f; US Biomax, Rockville, MD) were used. All human tissues on the TMAs were collected under HIPAA approved protocols by the supplier, US Biomax, Inc. (https://www.biomax.us/FAQs). Paraffin-embedded TMAs were deparaffinized and blocked for endogenous peroxidase activity with 0.3% hydrogen peroxide in methanol for 15 min. After rehydration, antigen retrieval was performed in 10 mM citrate buffer (pH 6.0, > 90 °C) for 20 min. Tissues were blocked for non-specific antibody binding with 5% Bovine Serum Albumin (BSA) in PBS for 30 min at room temperature, followed by overnight primary antibody incubation for PNPLA8 (Rabbit, 1:200, HPA020083, RRID: AB_1851849, Sigma) at 4 °C. Next, TMAs were incubated with biotin-conjugated secondary antibody for 30 min (Goat-anti-Rabbit, RRID: AB_3073814, Vectastain, Vectorlabs, Burlingame, CA), followed by incubation with avidin-streptavidin complex (Vectastain, Vectorlabs, Burlingame, CA) for 30 min. Sections were developed with 3,3′-diaminobenzidine (DAB; Dako, Glostrup, Denmark) and counterstained with hematoxylin. After dehydration, slides were mounted using Entellan (Merck Millipore, Burlington, Massachusetts, USA). The stains were digitized with an Aperio CS2 scanner (Leica Microsystems) using a 20 × magnification. Images were scanned using Aperio ImageScope (Version 12.3.3, Leica, Microsystems). The pixel H-score of PNPLA8-positive staining for each tissue core on the TMAs was calculated by using QuPath (Version 0.3.2, University of Edinburgh, UK). Briefly, tumor cells and stromal cells were classified by nuclear/cell area ratio. The classifier was further trained by manual cell annotations by a pathologist. H-scores of PNPLA8 staining of tumor cells were analyzed by the calculation of mean intensity of cytoplasm DAB.

### Cell viability

Cells (3 × 10^3^) were seeded onto a 96-well plate in 100 μL culture medium and incubated for 72 h at 37 °C. Then 10 μL of tetrazolium salt WST-1 (4-[3-(4-Iodophenyl)-2-(4-nitro-phenyl)-2H-5-tetrazolio]-1,3-benzene sulfonate) (Roche) was added into each well and incubated at 37 °C for 4 h. The absorbance was measured on an Epoch Microplate Spectrophotometer (BioTek, Winooski, US) at 450 nm.

### Cell migration

Cells (1 × 10^5^) were seeded on the upper chambers of an 8.0 μm pore size Transwell plate from Corning (#3422, Corning, USA). The lower chambers were supplemented with 600 μl DMEM and 10% FBS. After 24 h, invaded cells were fixed with methanol, stained with 0.1% crystal violet (Sigma-Aldrich, USA), and counted under a microscope (Olympus, USA).

### Measurement of reactive oxygen species (ROS)

Intracellular ROS levels were determined by using DCFDA-Cellular ROS Assay Kit (ab113851, Abcam) according to the manufacturer’s protocol. Briefly, cells (5 × 10^5^) were harvested and incubated with 1 μM DCFDA for 30 min at 37 °C. Then, the fluorescence was detected by Cytek Aurora flow cytometry (Cytek Biosciences, US) with excitation/emission at 485 nm/535 nm.

### Measurement of mitochondrial superoxide

Mitochondrial superoxide levels were determined by using MitoSOX Green mitochondrial superoxide indicator (M36005, Thermo Fisher Scientific) according to the manufacturer’s protocol. Briefly, cells (5 × 10^5^) were harvested and incubated with 1 μM MitoSOX Green for 30 min at 37 °C. Then, the fluorescence was detected by Cytek Aurora flow cytometry (Cytek Biosciences, US) with excitation/emission at 488 nm/510 nm.

### Eicosanoid extraction

Cell homogenate (200 uL in ddH_2_O) was spiked with 50 uL of eicosanoids heavy isotope internal standards mixture (10 ng/mL) followed by addition of ddH_2_O (750 uL) to make a total volume of 1 mL cell suspension. Eicosanoids from cell suspension (1 mL) were extracted using solid phase extraction (SPE) as described by Wang et al. with minor changes [26]. Briefly, Strata TM-X 33 µm polymeric reversed phase SPE columns (cat # 8B-S100-UBJ; Phenomenex, CA, US) were preconditioned with 3.0 mL of 100% methanol, followed by 3.0 mL of water. The samples (1 mL) were then loaded into the SPE columns and washed with 2 mL of ddH_2_O followed by 2.0 mL of 10% methanol to elute polar and semi-polar metabolites. Finally, eicosanoids were eluted from SPE with 1.5 mL of 100% methanol. The collected extracts were completely dried under a nitrogen evaporator (Organomation, MA, USA) and stored at − 80 °C until analysis.

### LC–MS/MS analysis of eicosanoids

LC–MS/MS analysis of eicosanoids was performed as described earlier [26] with some optimizations. Briefly, eicosanoids were separated on a C18 reverse-phase column 2.6 µm, 100 × 2.1 mm (Phenomenex, Torrance, CA, USA) employing a binary mobile phase gradient program (Eluate-A: ACN/water/acetic acid (60/40/0.02, v/v), and eluate-B: ACN/IPA (50/50, v/v)) using an Ultrafast Liquid Chromatography (UFLC) system (Shimadzu, Nakagyo-ku, Kyoto, Japan). The gradient elution for 13 min was as follows: 0.1–90% B (0.01–9.0 min); hold 90% B for 2 min (9.0–11.0 min); 90–0.1% B (11.00–13.00 min) at a constant flow rate of 0.4 mL/min. Eluted eicosanoids were introduced into hybrid quadrupole ion trap (API4000 QTRAP LC–MS/MS, AB Sciex, ON, Canada) mass spectrometer where individual eicosanoids were ionized in electrospray ionization (ESI) negative mode and acquired under multiple reaction monitoring (MRM) mode for quantification. Mass spectrometer source and analyzer parameters were optimized to get good signal for all eicosanoid species. Eicosanoid standard cocktail (Cayman Chemicals, MI, USA and Avanti Polar Lipids, AL, USA) was used to construct nine-point calibration curves (1, 10, 25, 50, 75, 100, 150, 200, and 500 ng/mL) by plotting the graph between area under the curve (AUC) response to the standards concentrations. These calibration curves were employed to the measured AUC for analytes in the extracted samples to calculate measured quantities in each sample. Instrument control and data acquisition were performed using Analyst (version 1.4.2, SCIEX Inc. Thornhill, Ontario, Canada), and data analysis was completed using MultiQuant software (version 2.0, SCIEX, Thornhill, ON, Canada).

### Prostaglandin E2 ELISA assay

Prostaglandin E2 levels of cell culture medium were determined by using a PGE2 ELISA Kit (ab133055, Abcam). Briefly, cells (2 × 10^4^) suspended in 200 μL culture medium were plated in 96-well plates. After 24 h, cell culture media were collected and centrifuged at 2000 rpm for 5 min. Then, the supernatants were collected for subsequent PGE2 analysis according to the manufacturer’s protocol. After incubation, para-Nitrophenylphosphate (pNpp) substrate was added to the ELISA plate, and samples were analyzed using an Epoch Microplate Spectrophotometer (BioTek, Winooski, US) at 405 nm.

### Volcano-plot analysis

The average intensities of individual lipids in the immortal human mammary epithelial cell lines MCF10A and MCF12A were calculated as controls. The average intensities of individual lipids in the TNBC cell lines MDA-MB-231, SUM159PT, Hs578T, MDA-MB-468 and SUM149PT were calculated as TNBC comparison group. Volcano plots of fold changes of individual lipids in the TNBC group compared with controls were conducted by using the SIMCA software version 14.1 (Umetrics, Sweden).

### S-plot analysis

Immortal human mammary epithelial cell lines MCF10A and MCF12A were assigned to control group. TNBC cell lines MDA-MB-231, SUM159PT, Hs578T, MDA-MB-468 and SUM149PT were assigned to TNBC group. The intensities of individual lipids of the control and the TNBC group were uploaded to the SIMCA software version 14.1 (Umetrics, Sweden) to conduct S-plot analysis. S-plot visualizes the covariance and the correlation structure between individual lipids and the predictive score of the predictive component. The confidence of individual lipids as a discriminant of variance increases with increasing numerical values on the y-axis and the size of the contribution increases with increasing numerical values on the x-axis. Individual lipids that were most up- or down-regulated in the TNBC group were selected at the cutoff value p(corr) ≥ 0.7 for upregulation (labeled as red dots) and ≤ -0.7 for downregulation (labeled as blue dots).

### Correlation analysis of protein expression and lipid levels

The densities of the Western Blot bands were analyzed by Image Laboratory software version 4.0.1 (Bio-Rad) and normalized by GAPDH intensity of each sample, which represented relative protein expression levels. Pearson’s correlation analyses of protein expression and lipid levels were conducted by using GraphPad Prism 5 software (La Jolla, CA, USA).

### Statistical analysis

Principal component analysis (PCA) and orthogonal partial least-squares discriminant analysis (OPLS-DA) were conducted by using SIMCA software version 14.1 (Umetrics, Sweden). MetaboAnalyst 5.0 (http://www.metaboanalyst.ca/) was used for performing clustering analysis and heatmap analysis. Venny 2.1 (https://bioinfogp.cnb.csic.es/tools/venny/index.html) was used for preparing Venn-diagrams. Gene set enrichment analysis (GSEA) was performed using GSEA software (version 4.2, San Diego, CA, USA) to analyze the correlation of PNPLA8 to hallmark gene sets according to the data of the TCGA breast cancer dataset. The Kaplan–Meier Plotter (http://www.kmplot.com/) breast cancer mRNA gene chip platform was used for generating Kaplan–Meier curves based on the specified gene expression levels for the reported patient survival rates. The probes selected for the analysis of each gene are indicated in their corresponding figures. Bar graph analyses were conducted by using GraphPad Prism 5 software (La Jolla, CA, USA). Differences between groups were evaluated using an unpaired two-tailed student’s t test. p values < 0.05 were considered significant.

## Results

### Lipid metabolic reprogramming in TNBC cells

**Fig. 1:**
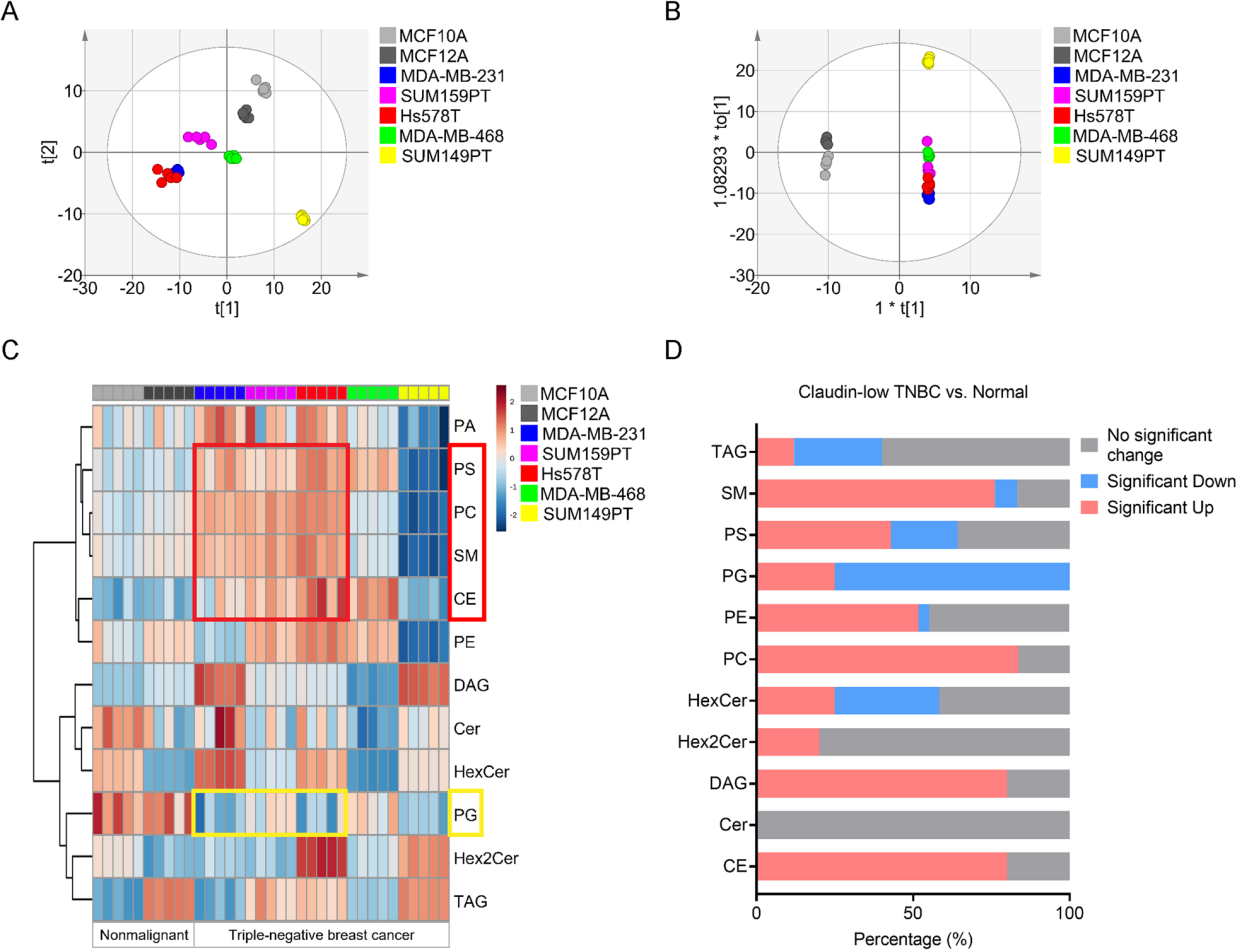
Lipid remodeling in triple-negative breast cancer cell lines. **A** PCA score scatter plot of lipid features in TNBC (MDA-MB-231, SUM159PT, Hs578T, MDA-MB-468, SUM149PT) and immortal human mammary epithelial cell lines (MCF10A, MCF12A). The x-axis and y-axis indicate the first principal component and second principal component, respectively. **B** OPLS-DA score scatter plot of TNBC cell lines compared to immortal human mammary epithelial cell lines. **C** Heatmap of log normalized abundance of lipid classes in TNBC and nonmalignant human mammary epithelial cell lines. Each cell line is represented by 5 biological replicates. Red boxes outline phospholipids increased in TNBC cells, and yellow boxes outline phospholipids decreased in TNBC cells. **D** The percentage of significantly upregulated, downregulated, and unchanged species of each lipid classes in Claudin-low TNBC cell lines (MDA-MB-231, SUM159PT and Hs578T) compared to immortal human mammary epithelial cell lines. p values < 0.05 were considered significant

We obtained comprehensive lipid profiles of five human TNBC cell lines (MDA-MB-231, SUM159PT, Hs578T, MDA-MB-468, SUM149PT) and two nonmalignant breast epithelial cell lines (MCF10A, MCF12A) as control cell lines by performing a large-scale lipidomic Triple TOF MS/MS^ALL^ approach in which 251 lipids were quantified across all the cell lines. Principle component analysis (PCA) of these 251 ion features showed clear clustering across all biological replicates for each cell line, providing proof of reliability in sample preparation and analysis (Fig. 1A). Supervised orthogonal partial least-squares discrimination analysis (OPLS-DA) of the same data revealed excellent separation of all TNBC cell clusters and the two control cell line clusters, demonstrating significant differences of lipid profiles between TNBC and control cell lines (Fig. 1B). However, as the only inflammatory cell line among the five tested TNBC cell lines, both PCA and OPLS-DA showed that the SUM149PT cluster was different from the other four TNBC cell lines. We classified the lipids into 12 categories and used Lipid Class-Wise Analysis to compare lipid alterations among cell lines. The heatmap indicates that the lipid phenotypes are more consistent in the three claudin low-TNBC cell lines (MDA-MB-231, SUM159PT, Hs578T) than the other TNBC cell lines (MDA-MB-468, SUM149PT). The heatmap shows a significant decrease in PG in all five TNBC cell lines and an increase in PS, PC, SM and cholesterol ester (CE) in the three claudin-low TNBC cell lines, relative to the control cell lines (Fig. 1C). Heatmaps of lipid species in each class are shown in Additional file 1: Fig. S1(A–K). Figure 1D shows the percentage of significantly upregulated and downregulated lipid species of each class in claudin-low TNBC cell lines. These results also indicate that most of the PG species are downregulated and PC, CE and SM levels are upregulated in claudin-low TNBC cell lines.

**Fig. 2:**
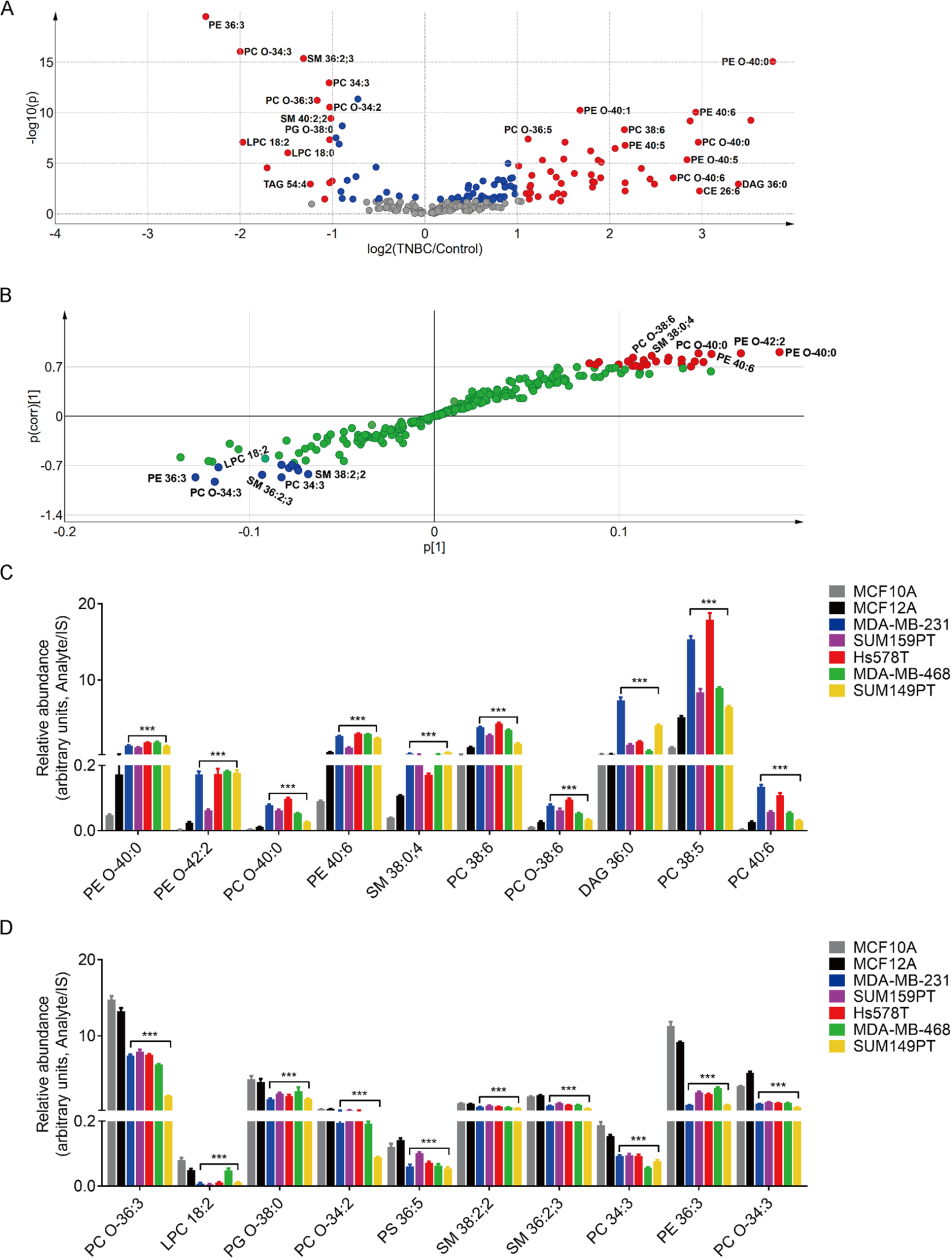
Critical lipid species dysregulated in triple-negative breast cancer cell lines. **A** Volcano plot of fold changes of individual lipids in TNBC cell lines (MDA-MB-231, SUM159PT, Hs578T, MDA-MB-468, SUM149PT) compared with immortal human mammary epithelial cell lines (MCF10A, MCF12A). Each sphere represents one ion feature. Blue spheres represent lipids with p < 0.05. Red spheres represent lipids with p < 0.05 and fold change (FC) > 2 or (FC) < 0.5. **B** S-plot comparing TNBC cell lines to immortal human mammary epithelial cell lines. Each green sphere represents one ion feature. Red and blue spheres represent significant upregulated or downregulated lipids, respectively, in TNBC cell lines compared with control cell lines. **C–D** Relative abundance of the top 10 of both upregulated (**C**) and downregulated (**D**) lipids selected by S-plot significance shown in (B) in TNBC cell lines compared with two immortal human mammary epithelial cell lines. PC/PE O-represents ether linked phospholipids. For hexosylceramide (HexCer)/Hex2Cer/SM x:y;z, x represents fatty acid carbon chain length, y represents number of double bonds on fatty acid moiety, and z represents oxygen molecules present on fatty acid moiety. ***p < 0.0001

To further identify specific dysregulated phospholipid species in TNBC, we used volcano plots to show the fold change of each lipid (x-axis) in TNBC cell lines *versus* control cell lines, with the corresponding statistical significance (y-axis) (Fig. 2A). Volcano plots show that, in TNBC cells, most of the significantly altered lipids were PCs and PEs (Fig. 2A, Additional file 1: Table S3). We then used s-plot to visualize the correlation between the lipids and their predictive scores to discriminate TNBC from the control cell lines. We set the cutoff value (y-axis) as 0.7 to select the most significantly dysregulated lipids. S-plot identified 24 upregulated (red) and 10 downregulated (blue) lipids in TNBC compared with control cells (Fig. 2B, Additional file 1: Table S4). The dysregulated lipids obtained by s-plot overlapped with significantly altered lipids identified by volcano plot. Figure 2C, D shows the relative concentrations of the top 10 of both upregulated (PE O-40:0, PE O-42:2, PC O-40:0, PE 40:6, SM 38:0;4, PC 38:6, PC O-38:6, DAG 36:0, PC 38:5, and PC 40:6) and downregulated lipids (PC O-36:3, LPC 18:2, PG O-38:0, PC O-34:2, PS 36:5, SM 38:2;2, SM 36:2;3, PC 34:3, PE 36:3, and PC O-34:3) from the s-plot analysis across all the cell lines. Taken together, our lipidomic analysis revealed significant phospholipid metabolic reprogramming in TNBC cells.

### PNPLA8 is upregulated in TNBC cell lines and correlates with poor outcome in breast cancer patients

**Fig. 3:**
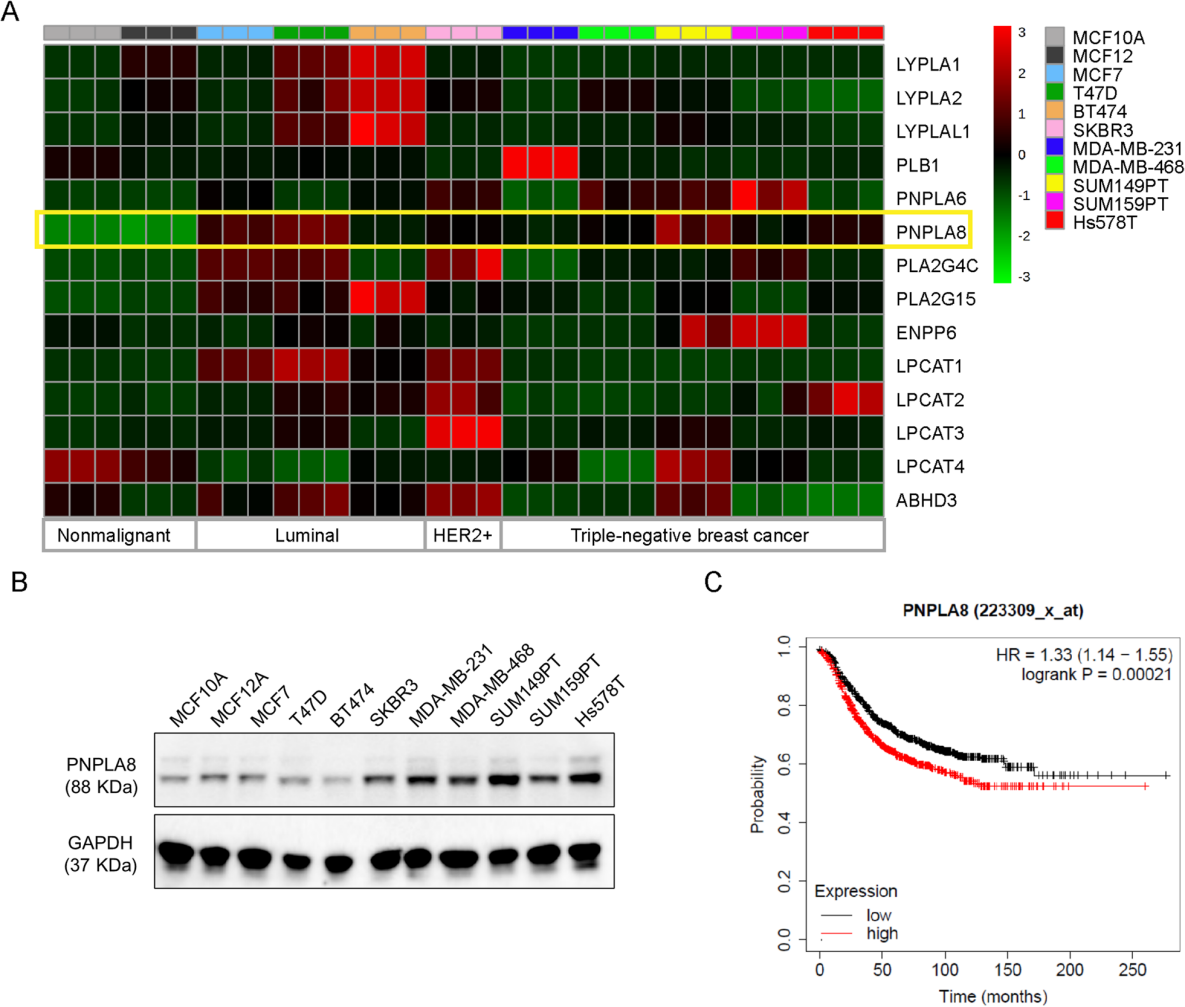
PNPLA8 expression levels in breast cancer cell lines and its correlation with breast cancer patient survival. **A** Heatmap of log normalized mRNA level of key genes involved in phospholipid metabolism in our panel of breast epithelial (MCF10A, MCF12A) and breast cancer cell lines (MCF7, BT474, SKBR3, MDA-MB-231, MDA-MB-468, SUM149PT, SUM159PT, Hs578T). Each cell line is represented by 3 biological replicates. The yellow box outlines PNPLA8 overexpression in breast cancer cells. **B** Protein expression level of PNPLA8 in a panel of breast cancer cell lines. GAPDH was used as a control to confirm equal loading of protein. **C** Relapse-free survival rates of breast cancer patients with low (n = 1019) or high (n = 1013) expression levels of PNPLA8. The survival curve was obtained from KM-plotter

The phospholipid composition of cell membranes is mainly maintained through a remodeling process of deacylation and reacylation referred to as the Lands cycle in which phospholipases hydrolyze glycerophospholipids to generate lysophospholipids, while lysophosphatidylcholine acyltransferases (LPCAT) catalyze the reacylation of lysophospholipids at the *sn-2* position [13]. We aimed to investigate the dysregulated key enzymes of phospholipid metabolism, including those in the Lands cycle, in TNBC cells. To screen for dysregulated enzymes which are specific to TNBC subtype, we have included luminal and HER2+ cell lines for comparison. qRT-PCR screening was performed to evaluate the mRNA expression levels of essential genes encoding LPCAT and enzymes both having phospholipase and lysophospholipase activity. This qRT-PCR screening showed that the mRNA levels of iPLAγ, also referred to as patatin-like phospholipase domain-containing lipase 8 (PNPLA8), cPLA2γ (PLA2G4C) and lysosomal phospholipase A2 (PLA2G15) were upregulated, and LPCAT4 was downregulated in a panel of breast cancer cell lines compared to nonmalignant breast epithelial cells (Fig. 3A). PNPLA8 is one of the nine PNPLAs (PNPLA1-9) known in humans [27]. Only PNPLA 6–9 hydrolyze phospholipids and are not well-studied in cancer [28]. We measured the mRNA levels of PNPLA 6–9, but only PNPLA6 and PNPLA8 were detected in our cell lines. Our results also showed that PNPLA8 protein levels were overexpressed in HER2+ (SKBR3) and TNBC (MDA-MB-231, MDA-MB-468, SUM159P, Hs578T) breast cancer cell lines compared to other cell lines (Fig. 3B). However, the trends of PNPLA8 mRNA and protein levels were not consistently matched in each tested breast cancer cell line. PNPLA8 mRNA levels were higher in MCF7 and T47D cell lines than those of most TNBC cell lines (Fig. 3A) while PNPLA8 protein levels were most significantly overexpressed in TNBC cell lines (Fig. 3B). The different trends could be caused by epigenetic or post-transcriptional regulation [29, 30]. Our results showed that higher mRNA levels of PNPLA8 in primary breast tumors were correlated with shorter relapse-free survival in breast cancer patients (Fig. 3C). LPCAT4 protein levels were downregulated in most of the breast cancer cell lines (Additional file 1: Fig. S2A), and lower mRNA levels of LPCAT4 were associated with longer relapse-free survival in breast cancer patients (Additional file 1: Fig. S2B). PLA2G4C was downregulated in the tested TNBC cell lines (Additional file 1: Fig. S2A), and higher PLA2G4C mRNA levels correlated with longer survival in breast cancer patients (Additional file 1: Fig. S2C). PLA2G15 was only expressed in a few breast cancer cell lines (Additional file 1: Fig. S2A), and no significant correlation was observed between PLA2G15 mRNA expression levels and breast cancer patient survival (Additional file 1: Fig. S2D). Overall, the mRNA and protein expressions of PNPLA8 and LPCAT4 were consistently dysregulated in the tested panel of breast cancer cell lines, and both gene expression levels were correlated with relapse-free survival in breast cancer patients.

Next, we performed correlation analysis of the protein levels of PNPLA8 and LPCAT4 with the detected lipid abundances in the tested panel of breast cancer cell lines, which was based on our Western Blot and lipidomic analysis results. This correlation analysis showed that the protein expression levels of PNPLA8 were positively correlated with seven phospholipid species (PE 40:4, PE 40:5, PE 40:6, PE O-40:0, PE O-42:2, PC O-36:5, and SM 38:0;4) and negatively correlated with three other phospholipid species (SM 38:2;2, SM 36:2;3, and PG O-38:0) (Additional file 1: Fig. S3). These ten significantly correlated lipid species displayed high predictive scores for discriminating TNBC from nonmalignant control cells (Additional file 1: Table S4). However, the three lipid species (PS 36:6, PE 46:0 and TAG 44:3) that significantly correlated with LPCAT4 protein expression levels (Additional file 1: Fig. S4) did not have high predictive scores for discriminating TNBC from nonmalignant mammary control cells (Additional file 1: Table S4). Based on these findings, we further pursued PNPLA8 for in depth molecular studies.

### PNPLA8 is upregulated in breast cancer, especially in TNBC tissues, and correlates with tumor grade, stage and lymph node metastasis

**Fig. 4:**
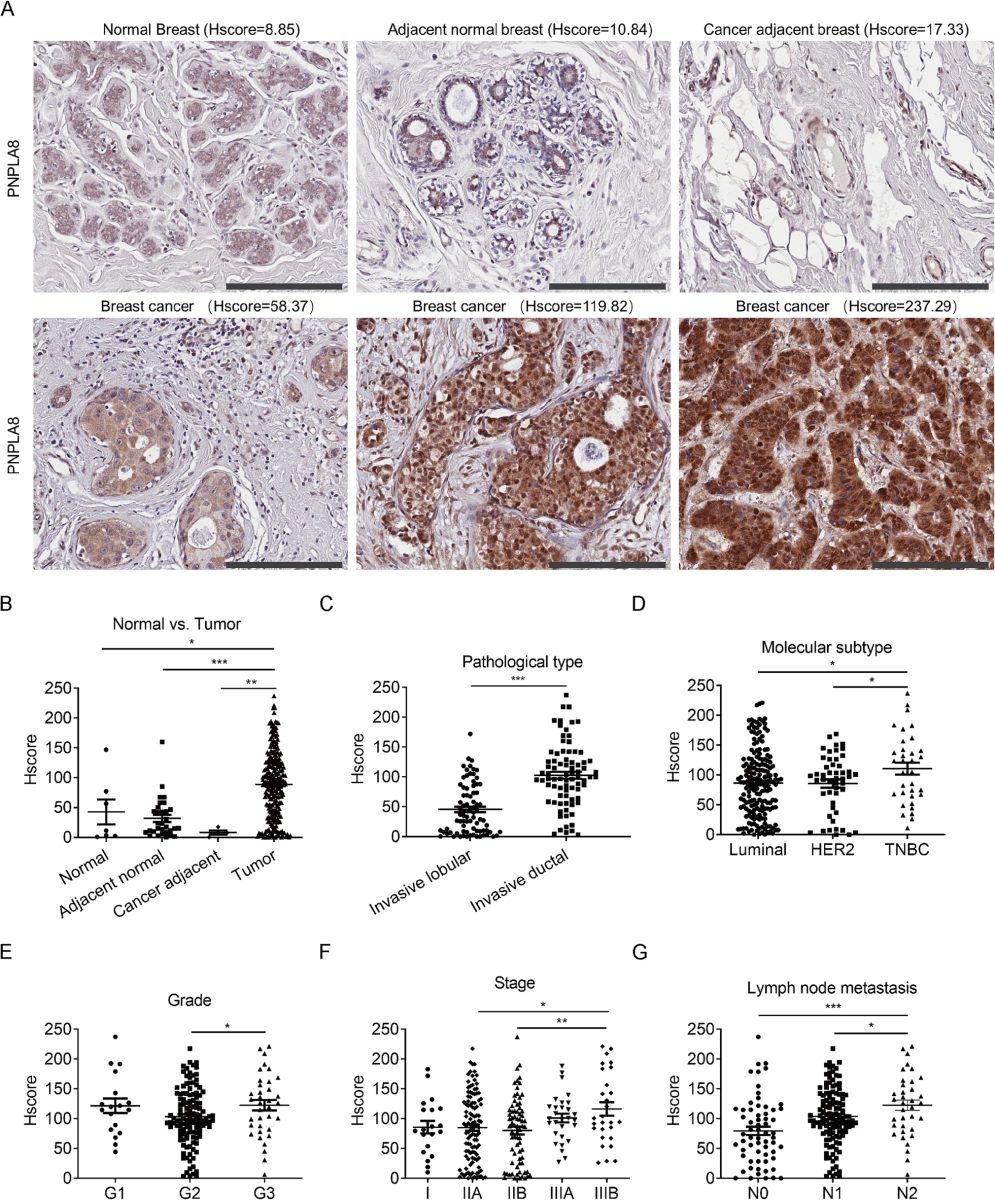
PNPLA8 expression levels in breast cancer tissues and its correlation with disease development. **A** Representative IHC staining of PNPLA8 expression from breast cancer TMAs. Scale bar = 200 μm. **B** PNPLA8 expression levels in normal breast tissues (n = 7), adjacent normal breast tissues (n = 31), cancer adjacent breast tissues (n = 4) and invasive breast cancer tissues (n = 280). **C** PNPLA8 expression levels in invasive lobular and invasive ductal breast cancer tissues. **D** PNPLA8 expression levels in tumor tissues of Luminal, HER2+ and TNBC subtypes. **E** PNPLA8 expression levels in histological grade 1 (G1), 2 (G2) and 3 (G3) breast cancer patients. **F** PNPLA8 expression levels in different TNM stages of breast cancer patients. **G** PNPLA8 expression levels in tumor tissues with no (N0), 1 ~ 3 (N1) and 4 ~ 9 (N2) lymph node metastases. *p < 0.05, ***p < 0.0001

To confirm the clinical significance of PNPLA8 in breast cancer, breast cancer tissue microarrays (TMAs) were subjected to immunochemistry (IHC) to evaluate PNPLA8 expression levels in breast cancer patients compared to healthy subjects. The IHC scores for PNPLA8 revealed that PNPLA8 was overexpressed in invasive breast cancer tissues (n = 280) compared to normal breast tissues (n = 7), adjacent normal breast tissues (n = 31) or cancer adjacent breast tissues (n = 4) (Fig. 4A, B), which is consistent with the results of PNPLA8 protein expression levels from the Clinical Proteomic Tumor Analysis Consortium (CPTAC) (https://ualcan.path.uab.edu/analysis-prot.html) breast cancer dataset measured by proteomics (Additional file 1: Fig. S5A) [31, 32]. Moreover, PNPLA8 levels were higher in invasive ductal breast cancer tissues than those in invasive lobular breast cancer tissues (Fig. 4C). Tumor tissues of the TNBC subtype showed higher PNPLA8 levels compared to luminal and HER2+ breast cancer tissues (Fig. 4D). The CPTAC breast cancer dataset also showed that PNPLA8 protein levels in TNBC were higher than those in normal breast tissues and other breast cancer subtypes, although there was no statistical significance between TNBC and other subtypes owing to insufficient sample size for the TNBC group (Additional file 1: Fig. S5B). In addition, PNPLA8 levels were increased in histological grade 3 (G3) breast cancer patients as compared to grade 2 (G2) patients (Fig. 4E) and were also increased in TNM stage IIIB breast cancer patients as compared to stage IIB and IIA patients (Fig. 4F). Moreover, PNPLA8 expression levels in breast cancer tissues were higher in patients with more significant lymph node metastasis (Fig. 4G). However, we did not find any significant changes of PNPLA8 expression levels in tumor tissues with different tumor size, ER, PR and HER2 expression status (Additional file 1: Fig. S6A–D). Taken together, our results show that PNPLA8 is overexpressed in breast cancer tissues and correlates with pathological classification, molecular subtype, and tumor development.

### PNPLA8 maintains oxidative stress balance and promotes cell viability and migration in TNBC cells

**Fig. 5:**
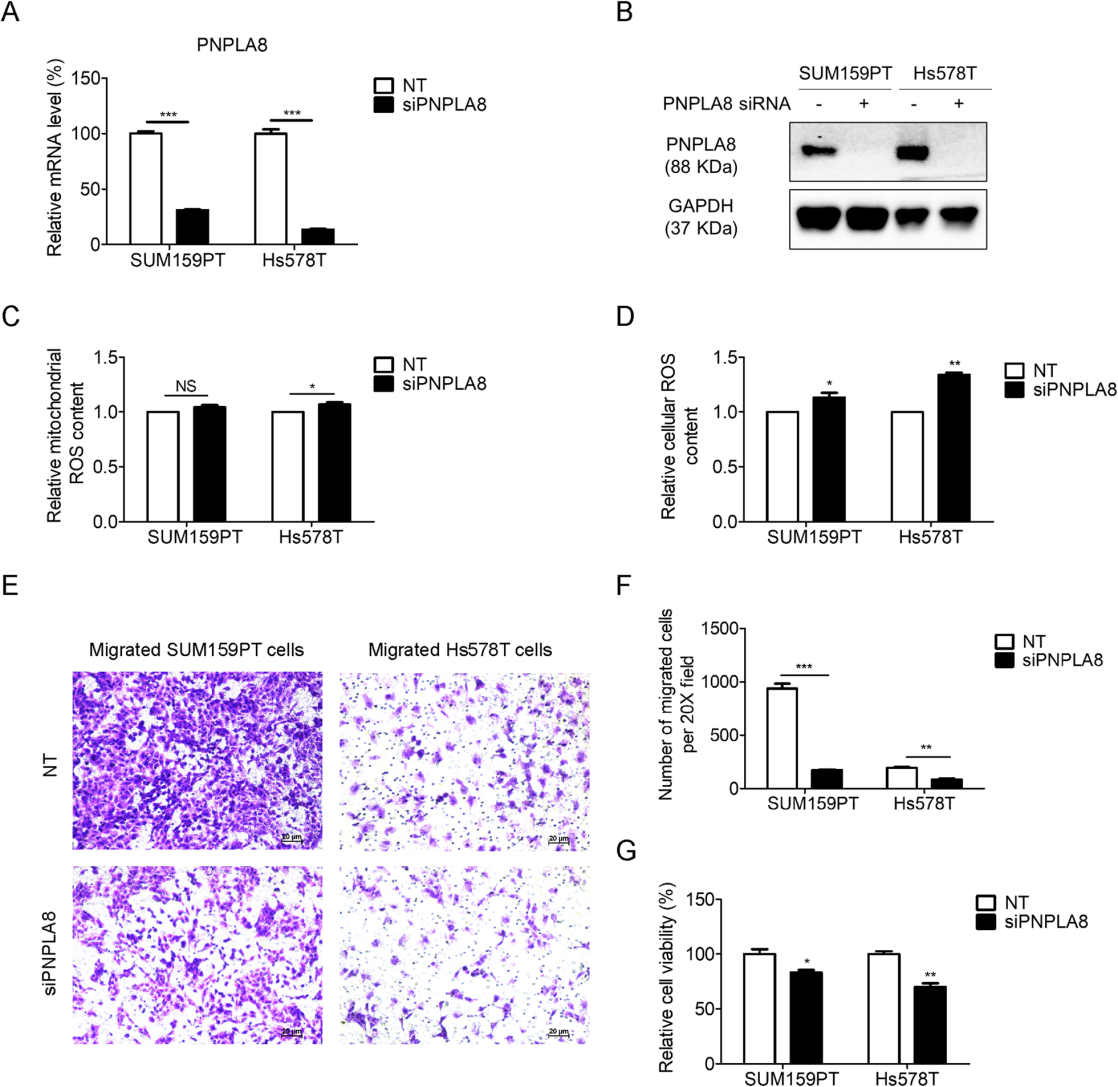
Silencing of PNPLA8 increases cellular reactive oxygen species levels and decreases cell viability and cell migration. **A** Real-time PCR showing mRNA levels of PNPLA8 in SUM159PT and Hs578T cell lines treated with PNPLA8 siRNA or scrambled siRNA. Data are represented as mean values ± S.D. of 3 independent experiments. **B** Protein expression levels of PNPLA8 in SUM159PT and Hs578T cell lines treated with PNPLA8 siRNA or scrambled siRNA. GAPDH was used as a control to confirm equal loading of protein. **C** Flow cytometry analysis of mitochondrial reactive oxygen species (ROS) using MitoSOX in SUM159PT and Hs578T cell lines treated with PNPLA8 siRNA or scrambled siRNA as controls. Values represent MitoSOX intensity mean values ± S.D. of 3 independent experiments. **D** Flow cytometry analysis of reactive oxygen species (ROS) using DCFDA in SUM159PT and Hs578T cell lines treated with PNPLA8 siRNA or scrambled siRNA as controls. Values represent DCFDA intensity mean values ± S.D. of 3 independent experiments. **E** Representative images of migrated cells of SUM159PT and Hs578T cell lines treated with PNPLA8 siRNA or scrambled siRNA as control. **F** Number of migrated cells of SUM159PT and Hs578T cell lines treated with PNPLA8 siRNA or scrambled siRNA as control. Migrated cells were counted using Image J. Each cell line is represented by 3 biological replicates. **G** Cell viability of SUM159PT and Hs578T cell lines treated with PNPLA8 siRNA or scrambled siRNA as control. Each cell line is represented by 3 biological replicates. *p < 0.05, **p < 0.001, ***p < 0.0001

As the two claudin-low TNBC cell lines, SUM159PT and Hs578T both have high expression levels of PNPLA8 and display similar characteristics of phospholipid metabolism, we selected these two cell lines for the following studies. We silenced the expression of PNPLA8 by using pooled PNPLA8 siRNA. The mRNA and protein levels of PNPLA8 decreased significantly following treatment with PNPLA8 siRNA in both TNBC cell lines, demonstrating the effectiveness of the knockdown (Fig. 5A, B). As PNPLA8 has been reported to localize to mitochondrial membranes and peroxisomes and is associated with the integrity of mitochondrial membranes and anti-mitochondrial oxidative stress [33–35], we measured both mitochondrial reactive oxygen species (ROS) and general ROS levels in TNBC cell lines following PNPLA8 siRNA silencing as compared to scrambled siRNA controls using ROS-specific fluorescent probes. Our results show that silencing of PNPLA8 slightly increased mitochondrial ROS levels in Hs578T cells, but not in SUM159PT cells (Fig. 5C). Nevertheless, silencing of PNPLA8 increased total cellular ROS levels in both TNBC cell lines (Fig. 5D). These data indicate that PNPLA8 is crucial in maintaining the overall balance of ROS in TNBC cells, while mitochondrial ROS regulation may be subject to other mechanisms in addition to PNPLA8. As shown in Fig. 4G, PNPLA8 protein levels in breast cancer tissues are correlated with lymph node metastasis. Therefore, we measured cell migration and cell viability in TNBC cells following PNPLA8 siRNA silencing. The results demonstrate that knockdown of PNPLA8 decreased cell migration and cell viability in TNBC cell lines (Fig. 3E–G). Taken together, our data show that PNPLA8 is essential in maintaining the balance of cellular ROS and increasing cell viability and migration in TNBC cells.

### PNPLA8 mediates phospholipid metabolic reprogramming in triple-negative breast cancer cells

**Fig. 6:**
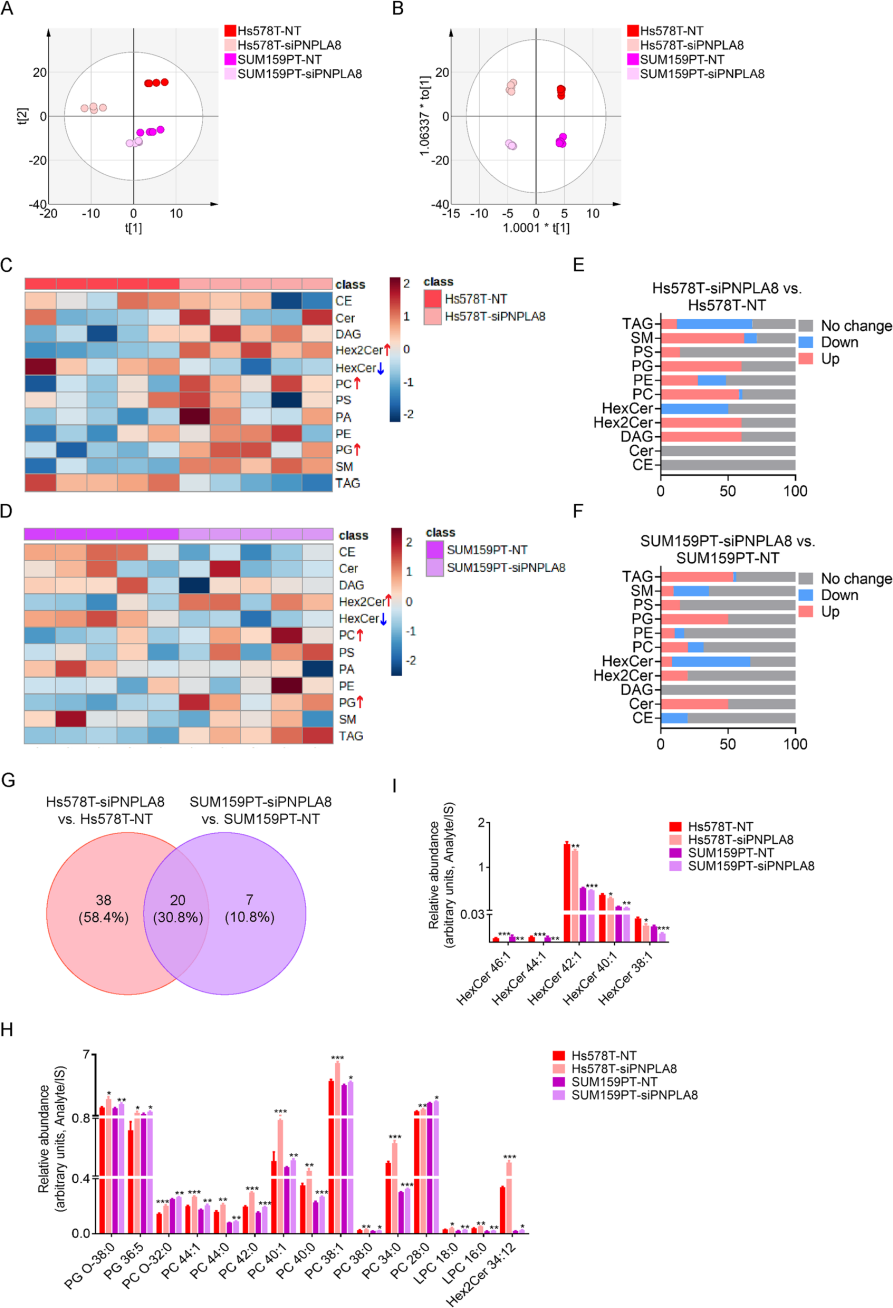
Silencing of PNPLA8 disrupts phospholipid remodeling. **A** PCA score scatter plot of lipid features in SUM159PT and Hs578T cell lines treated with PNPLA8 siRNA or scrambled siRNA as control. The x-axis and y-axis indicate the first principal component and second principal component, respectively. **B** OPLS-DA score scatter plot of ion features in SUM159PT and Hs578T cell lines treated with PNPLA8 siRNA or scrambled siRNA as control. **C** Heatmap of log normalized abundance of lipid classes in PNPLA8 siRNA-treated Hs578T cells *versus* scrambled siRNA-treated Hs578T control cells. Each siRNA treatment group is represented by 5 biological replicates. Blue boxes outline phospholipids decreased in PNPLA8 siRNA-treated Hs578T cells. Red boxes outline phospholipids increased in PNPLA8 siRNA-treated Hs578T cells. **D** Heatmap of log normalized abundance of lipid classes in PNPLA8 siRNA-treated SUM159PT cells *versus* scrambled siRNA-treated SUM159PT control cells. Each siRNA treatment group is represented by 5 biological replicates. Blue boxes outline phospholipids decreased in PNPLA8 siRNA-treated SUM159PT cells. Red boxes outline phospholipids increased in PNPLA8 siRNA-treated SUM159PT cells. **E–F** Percentage of significantly upregulated, downregulated, and unchanged species of each lipid classe in PNPLA8 siRNA-treated Hs578T (**E**) and SUM159PT (**F**) cells versus their corresponding scrambled siRNA-treated control cells. p values < 0.05 were considered significant. **G** Venn-diagram showing the lipids which were consistently increased or decreased in PNPLA8 siRNA-treated SUM159PT (purple circle) and Hs578T cell lines (pink circle) compared with scrambled siRNA-treated SUM159PT and Hs578T cell lines. **H** Relative abundance of the 15 lipids which increased following PNPLA8 siRNA treatment as obtained from the Venn-diagram in (**G**). **I** Relative abundance of the 5 lipids which decreased following PNPLA8 siRNA treatment as obtained from the Venn-diagram in (**G**). *p < 0.05, **p < 0.001, ***p < 0.0001

We measured the changes of lipid profiles in PNPLA8-silenced SUM159PT and Hs578T cells using Triple TOF lipidomic profiling. Both the PCA and OPLS-DA demonstrated significant separation between PNPLA8 siRNA silenced cells and scrambled siRNA control cells (Fig. 6A, B). Lipid Class-Wise Analysis showed that PC, PG and Hex2Cer were increased and hexosylceramide (HexCer) was decreased in both PNPLA8-silenced Hs578T and SUM159PT cells (Fig. 6C, D). Figure 6E, F shows the percentage of significantly upregulated and downregulated lipid species of each class in PNPLA8-silenced TNBC cell lines, compared with the respective control cells. The results show that all PGs, Hex2Cers and most PCs and PEs were increased, while most of HexCers were decreased in both PNPLA8-silenced TNBC cell lines. Intriguingly, most TAGs were decreased and most SMs were increased in PNPLA8-silenced Hs578T cells (Fig. 6C, E). However, the trends of TAGs and SMs were opposite in PNPLA8-silenced SUM159PT cells (Fig. 6D, F), which may be due to differing preferences of using TAG versus SM as a substitute source of PG, Hex2Cers, PC and PE in the two cell lines. Among the above dysregulated phospholipid classes, there were 52 upregulated and 6 downregulated lipids in Hs578T-siPNPLA8 cells (Additional file 1: Table S5), and 19 upregulated and 8 downregulated lipids in SUM159PT-siPNPLA8 cells, compared with the respective control cells (Additional file 1: Table S6). There were 15 upregulated lipids and 5 downregulated lipids in both PNPLA8-silenced Hs578T and SUM159PT cells (Fig. 6G). The upregulated lipids following silencing of PNPLA8 were PC, LPC, PG and Hex2Cer species (Fig. 6H). The downregulated lipids following silencing of PNPLA8 were predominantly HexCer species (Fig. 4I). As GPC is a product of LPC breakdown, we measured GPC levels in PNPLA8-silenced cells by high-resolution ^1^H MRS. The results showed that GPC concentrations decreased significantly in both PNPLA8-silenced Hs578T and SUM159PT cells (Additional file 1: Fig. S7). These results clearly show that silencing of PNPLA8 blocks the breakdown of PC, LPC, PG and Hex2Cer in TNBC cells, which may lead cancer cells to use HexCer, TAG or SM as substitute sources of phospholipid for energy or signal transduction.

### PNPLA8 increases arachidonic acid and eicosanoid levels in TNBC cells

**Fig. 7:**
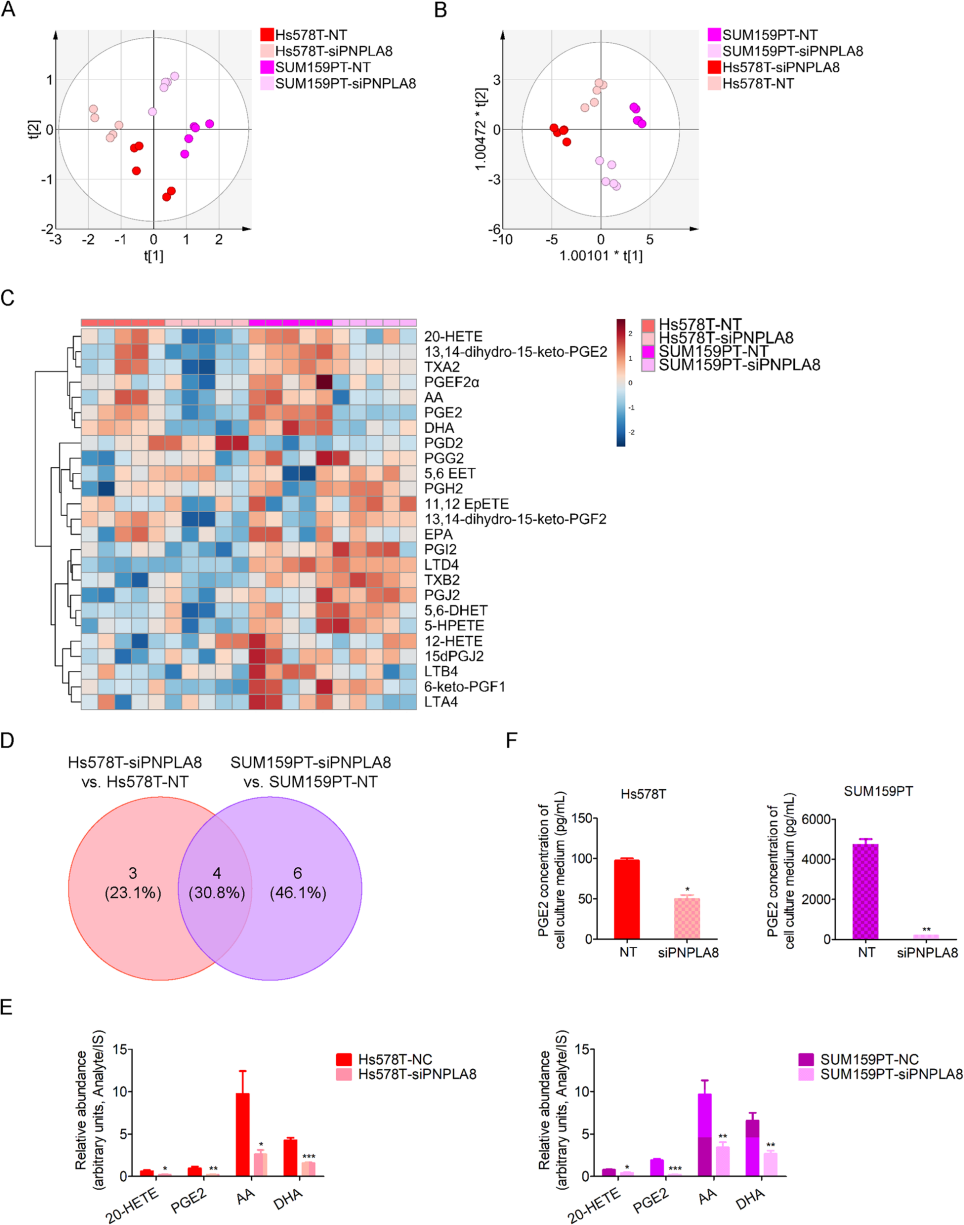
Silencing of PNPLA8 decreases cellular arachidonic, eicosanoids and secreted PGE2 levels. **A** PCA score scatter plot of eicosanoids in SUM159PT and Hs578T cell lines treated with PNPLA8 siRNA or scrambled siRNA as control. The x-axis and y-axis indicate the first principal component and second principal component, respectively. **B** OPLS-DA score scatter plot of eicosanoids in SUM159PT and Hs578T cell lines treated with PNPLA8 siRNA or scrambled siRNA as control. **C** Heatmap of log normalized abundance of eicosanoids in SUM159PT and Hs578T cell lines treated with PNPLA8 siRNA or scrambled siRNA as control. Each cell line is represented by 5 biological replicates. **D** Venn-diagram indicating the number of eicosanoids which were consistently increased or decreased following PNPLA8 siRNA treatment in both SUM159PT and Hs578T cell lines. **E** Relative abundance of eicosanoids from the Venn-diagram in (**D**). **F** PGE2 levels in culture media of SUM159PT and Hs578T cell lines treated with PNPLA8 siRNA or scrambled siRNA as control. Each cell line is represented by 3 biological replicates. *p < 0.05, **p < 0.001, ***p < 0.0001

PNPLA8 predominantly catalyzes the cleavage of membrane phospholipids to release arachidonic acid (AA) [36]. AA generates eicosanoids, including prostanoids, leukotrienes, hydroxyeicosatetraenoic acids (HETEs), epoxyeicosatrienoic acids (EETs) and hydroperoxyeicosatetraenoic acids (HPETEs), which play crucial roles in cancer and chronic inflammation [37]. We simultaneously measured cellular eicosanoids and their upstream precursor levels (total of 25) using LC–MS/MS analysis in SUM159PT and Hs578T cells silenced with PNPLA8 siRNA compared with scrambled siRNA control cells. A significant difference was observed between PNPLA8-silenced and control cells based on the resulting PCA and OPLS-DA plots from our LC–MS/MS eicosanoid panel measurements (Fig. 7A, B). The heatmap shows the relative abundance of all eicosanoids which we detected by LC–MS/MS (Fig. 7C). The cellular levels of nine eicosanoids and their precursors (13,14-dihydro-15-keto-PGF2α, 20-HETE, PGE2, leukotriene (LT) LTA4, LTB4, thromboxane (TX) TXA2, AA and docosahexaenoic acid (DHA)) were significantly decreased, and cellular levels of thromboxane 2 (TXB2) were increased in SUM159PT-siPNPLA8 cells compared to SUM159PT-NT control cells. Five eicosanoids (13,14-dihydro-15-keto-PGE2, 20-HETE, PGE2, PGI2, 6-keto-PGF1α) and two precursors (AA and DHA) displayed lower levels in Hs578T-siPNPLA8 cells compared with Hs578T-NT control cells. There were two eicosanoids (20-HETE and PGE2) and two precursors (AA and DHA) which were decreased in both PNPLA8-silenced TNBC cell lines compared to their respective controls (Fig. 7D, E). Since eicosanoids are frequently secreted into the extracellular space, we measured the PGE2 concentration in cell culture medium following silencing with PNPLA8 siRNA as compared to control. Our results show that PGE2 levels in cell culture medium were significantly decreased following silencing of PNPLA8 (Fig. 7F). Taken together, our results demonstrate that PNPLA8 regulates AA metabolism and the production of eicosanoids, specifically PGE2 and 20-HETE, in TNBC cells.

### PNPLA8 activates the PI3K/Akt/GSK3β and MAPK pathways in TNBC cells

**Fig. 8:**
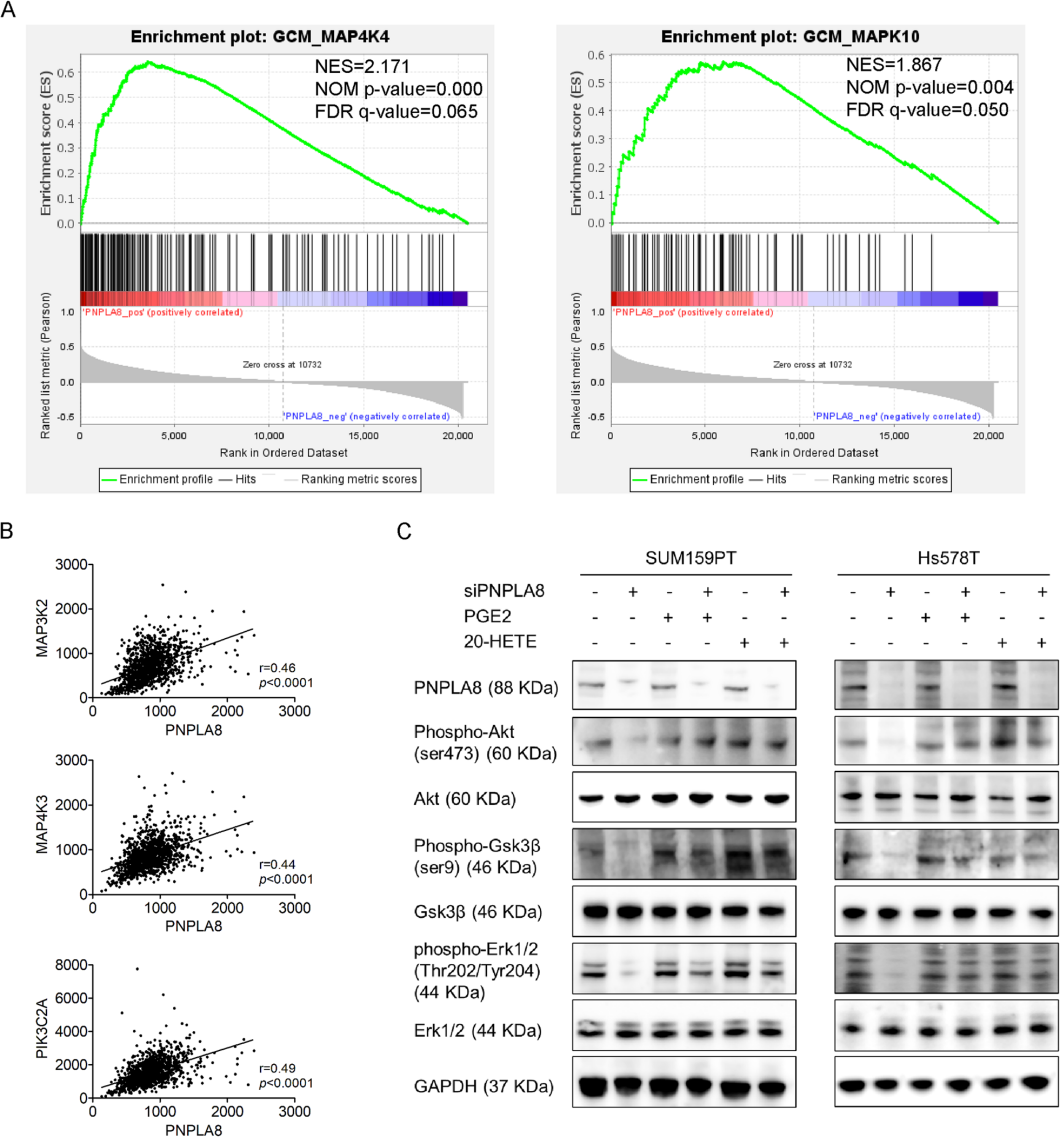
Silencing of PNPLA8 suppresses the PI3K/Akt/GSK3β and MAPK pathways. **A** GSEA results showed that MAP4K4 and MAPK10 pathways were enriched in the PNPLA8-positive cluster within the TCGA breast cancer data sets. NES, normalized enrichment score; NOM, normalized; FDR, false discovery rate. **B** Correlation analysis of MAP3K2, MAP4K3 and PIK3C2A with PNPLA8 mRNA levels in TCGA breast cancer tissues. **C** Protein expression levels of PNPLA8, phospho-Akt (ser473), Akt, phospho-Gsk3β (ser9), phospho-Erk1/2 (Thr202/Tyr204), Erk1/2 in SUM159PT and Hs578T cell lines treated with PNPLA8 siRNA or scrambled siRNA combined with the treatment of PGE2 (1 μM) or 20-HETE (10 nM) for 48 h. GAPDH was used as loading control

Prostaglandins and leukotrienes have prominent roles in promoting tumor progression through various signaling pathways [37]. To explore crucial signaling pathways associated with PNPLA8, we performed a Gene Set Enrichment Analysis (GSEA) using the data from The Cancer Genome Atlas (TCGA) breast cancer patient dataset [38]. GSEA found that PNPLA8 expression was positively correlated with mitogen-activated protein kinase kinase kinase kinase 4 (MAP4K4) and mitogen-activated protein kinase 10 (MAPK10) pathways (Fig. 8A). PNPLA8 correlation analysis based on the TCGA breast cancer data set showed that PNPLA8 was positively correlated with mitogen-activated protein kinase kinase kinase 2 (MAP3K2), mitogen-activated protein kinase kinase kinase kinase 3 (MAP4K3) and Phosphatidylinositol-4-Phosphate 3-Kinase Catalytic Subunit Type 2 Alpha (PIK3C2A), the key molecules of the MAPK and PI3K pathways (Fig. 8B) which are frequently activated in human cancers and are interconnected predominantly by sharing upstream receptors and joint downstream targets [39]. Based on the results of our GSEA and correlation analysis, we measured the protein levels of the key molecules involved in the MAPK and PI3K pathways in PNPLA8-silenced SUM159PT and Hs578T cells with the treatment of PGE2 or 20-HETE supplementation in cell culture medium. Our results show that the protein levels of phospho-AKT (ser473), phospho-GSK3β and phospho-Erk1/2 (Thr202/Tyr204) were significantly decreased in PNPLA8-silenced TNBC cells compared to controls and were recovered following PGE2 or 20-HETE supplementation (Fig. 8C), which clearly demonstrated that the PI3K and MAPK signaling pathways were deactivated upon PNPLA8 silencing through the suppression of PGE2 and 20-HETE production.

## Discussion

Metabolic reprogramming is a hallmark of cancer and promises to provide urgently needed targets for therapeutic intervention of TNBC to overcome current limitations in targeted TNBC treatment [10]. Our lipidomics analysis clearly demonstrated dysregulated phospholipid metabolism in TNBC cell lines. Gene screening and TMA studies showed for the first time that PNPLA8 expression is significantly elevated in TNBC cell lines and tissues and correlated with breast cancer patient survival, pathological classification, histological grade, TNM stage and lymph node metastasis. We found that the claudin-low TNBC cell lines (MDA-MB-231, SUM159PT and Hs578T) exhibited a similar phospholipid profile with a consistent increase in PCs, PSs, SMs and CEs which was not observed in the other two TNBC cell lines (MDA-MB-468, SUM149PT). As the claudin-low subtype is the most aggressive TNBC subtype, it could have a more representative phospholipid metabolism than other subtypes. Therefore, we selected two of the three claudin-low TNBC cell lines (SUM159PT and Hs578T) as cell models and further studied the function and mechanisms of PNPLA8 in TNBC.

PNPLA8 was reported to localize to endoplasmic reticulum, mitochondrial membranes and peroxisomes and hydrolyze oxidized phospholipids from biological membranes to maintain membrane integrity and eliminate oxidative stress [40–43]. PNPLA8 gene deficiency causes mitochondriopathy, myocadial dysfunction, neurodegeneration, metabolic syndrome and thrombosis in mice [36]. The role of PNPLA8 in cancer development is not well studied. Only one study so far has shown that PNPLA8 is overexpressed in human colorectal cancer tissues and promotes azoxymethane-induced colon carcinogenesis. Our data showed that silencing of PNPLA8 significantly reduced cell viability and cell migration, and increased total cellular ROS levels in TNBC cells, further linking high PNPLA8 levels to TNBC progression.

PNPLA8 has PLA1, PLA2 and lysophospholipase activities and preferentially hydrolyzes phosphatidylcholine and phosphatidylethanolamine species containing polyunsaturated fatty acids including arachidonic acid (AA) [36]. A recent study also found decreased hepatic GPC and choline levels in PNPLA8-deficient mice [44]. Our large-scale lipidomic analyses and ^1^H MRS analysis showed that silencing of PNPLA8 increased PC, LPC, PG, and Hex2Cer levels, while decreasing GPC levels in TNBC cells, which provides solid evidence that PNPLA8 mediates phospholipid remodeling in TNBC partly by promoting PC metabolism.

In addition to PCs, PGs were decreased in all TNBC cell lines as compared with nonmalignant breast epithelial cell lines. PGs are distributed in mitochondria as precursors of cardiolipin synthesis and were reported to suppress inflammation through Toll-like receptors [45]. Considering this pathway, the decrease in PGs in TNBC cells may also be associated with an activation of inflammatory pathways in TNBC. Our study showed that PG O-38:0 was one of the top 10 downregulated lipids that have a high predictive score for discriminating TNBC from nonmalignant breast epithelial cell lines, which indicates that PG O-38:0 could be a potential lipid biomarker for TNBC. PG O-38:0 was decreased in TNBC cell lines and positively correlated with PNPLA8 protein levels, while silencing of PNPLA8 increased PG O-38:0 level in TNBC cells, indicating a regulatory role of PNPLA8 for PG O-38:0. Further supporting a link between PGs, COX-2, and inflammation, a recent study in macrophage-like cells indicated that PG (18:1)_1_ and PG (18:2)_2_ supplementation inhibited the mRNA expression of COX-2 in macrophage-like cells [18]. Future studies should further clarify if PNPLA8 and/or PG O-38:0 levels affect the expression or activity of cyclooxygenases (COXs), lipoxygenases (LOXs) or cytochrome P450 (CYP) monooxygenases. Our study is, to the best of our knowledge, the first report that causally links PNPLA8 with phospholipid remodeling in TNBC. In the future, we will measure PC, LPC and PG species in various breast cancer tissues from patients and analyze their correlation with PNPLA8 expression to further determine their potential value in TNBC diagnosis and treatment.

Here, we have reported for the first time that PNPLA8 was upregulated in TNBC cells and silencing of PNPLA8 decreased AA in TNBC cells. AA is metabolized to eicosanoids through three major pathways: the COX, LOX and CYP monooxygenase pathways [46]. Prostaglandins and thromboxanes (TXs) are the products of the COX pathway. PGE2 is the most abundant prostaglandin in cancers and plays a crucial role in tumorigenesis [47]. Our study revealed that silencing of PNPLA8 decreased both cellular and extracellular PGE2 levels in TNBC cell lines, which is consistent with a recent study showing that overexpression of PNPLA8 increased AA release and PGE2 production in colon cancer cells [48]. We also showed that silencing of PNPLA8 decreased 20-HETE levels in TNBC cells. 20-HETE is one of the products of the CYP monooxygenase pathway and is currently under consideration as novel therapeutic target to inhibit breast cancer metastasis [49, 50]. The decreases of AA, PGE2 and 20-HETE in PNPLA8-silenced TNBC cells support that high PNPLA8 levels in TNBC could drive breast cancer progression through the activation of the arachidonic acid cascade.

Our analysis of the TCGA breast cancer patient dataset revealed that PNPLA8 is associated with the PI3K and MAPK signaling pathway in breast cancer tissues. We clearly showed in our study that PNPLA8 is crucial in the activation of the PI3K and MAPK pathways in TNBC cell lines through the regulation of PGE2 and 20-HETE levels. Consistent with our study, both PGE2 and 20-HETE were shown to activate the epidermal growth factor receptor (EGFR)/PI3K/Akt signaling pathway in colorectal cancer and renal epithelial cells, respectively [51, 52], and stimulated the activation of the PI3K/Akt, MAPK/Erk or GSK3β/β-catenin pathway through binding of prostaglandin E2 receptors (EP)1-4 or G protein-coupled receptors (GPCR), respectively [37, 53].

In addition to PNPLA8, we also detected LPCAT4 mRNA and protein levels to be downregulated in breast cancer cell lines. PNPLA8 hydrolyzes glycerophospholipids to generate lysophospholipids, whereas LPCAT4 catalyzes the reacylation of lysophospholipids at the *sn-2* position, participating in the remodeling process of deacylation and reacylation referred to as the Lands cycle [13]. Upregulation of PNPLA8 and downregulation of LPCAT4 in TNBC cells as reported in our study point toward abnormal phospholipid turnover and active phospholipid degradation in TNBC cells.

**Fig. 9:**
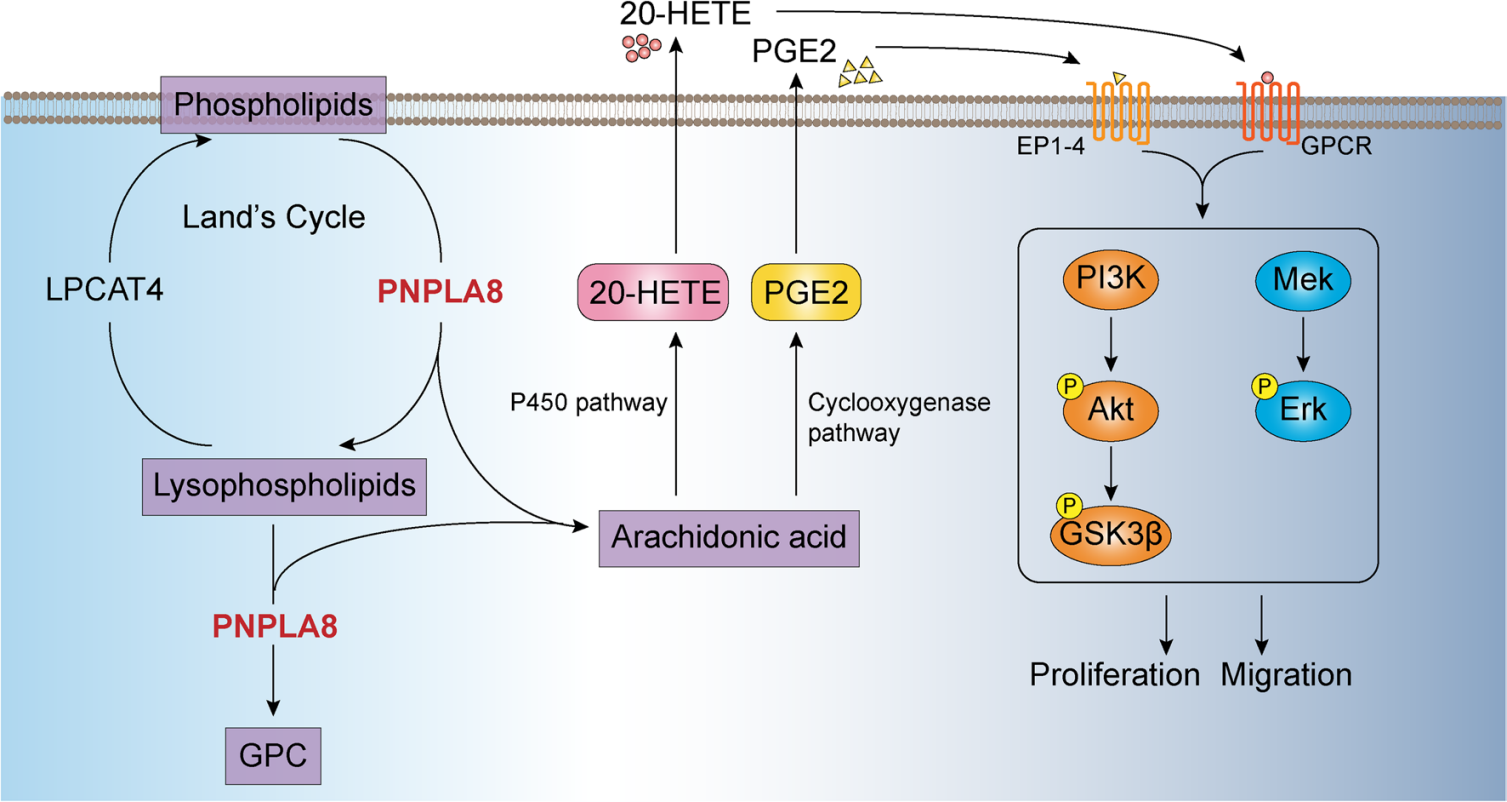
Proposed mechanism of PNPLA8 in the regulation of phospholipid metabolism resulting in increased migration and proliferation in TNBC through the activation of PI3K/Akt/GSK3β and MAPK pathways. Our data suggest that the upregulation of PNPLA8 and downregulation of LPCAT4 promotes the hydrolysis of phospholipids. Acting as both phospholipase and lysophospholipase, PNPLA8 mediates the production of lysophospholipids and GPC, releasing arachidonic acid, leading to elevated PGE2 and 20-HETE from arachidonic acid through the cyclooxygenase and cytochrome P450 pathways, respectively. Secreted PGE2 and 20-HETE can bind to prostaglandin E2 receptors (EP) and G protein-coupled receptors (GPCR), respectively, then activate the PI3K/Akt and MAPK/Erk signaling pathways, promoting cell proliferation and migration in triple-negative breast cancer

Mechanistically, our data support that the upregulation of PNPLA8 and downregulation of LPCAT4 promotes the hydrolysis of phospholipids and generates arachidonic acid and lysophospholipids, leading to elevated PGE2 and 20-HETE from arachidonic acid through the cyclooxygenase and cytochrome P450 pathways, respectively (Fig. 9). Secreted PGE2 and 20-HETE bind to prostaglandin E2 receptors (EP) and GPCR, respectively, then activate the PI3K/Akt and MAPK/Erk signaling pathways, promoting cell proliferation, migration and anti-oxidative stress in triple-negative breast cancer.

The translational relevance of our study is twofold. First, we have identified PG O-38:0 as a novel lipid biomarker of TNBC cells which could be translated to the clinic for determining surgical margins during breast-conserving breast tumor surgery using emerging intra-operative surgical mass spectrometry technologies for clinical margin detection with the iKnife [54] and the mass spec pen [55]. Second, we have discovered that PNPLA8 is a key master regulator of phospholipid reprogramming and eicosanoid signaling induced proliferation and migration in triple-negative breast cancer, which could be translated to the clinic as a novel diagnostic immunohistochemistry marker. PNPLA8 could also be developed as a new treatment target for TNBC, which currently has few targeted treatment options available.

## Conclusions

We show that PNPLA8 is upregulated in TNBC cells and promotes phospholipid remodeling, proliferation, antioxidation and migration of TNBC cells. PNPLA8 activates the arachidonic acid cascade and the production of PGE2 and 20-HETE, which in turn activate the PI3K/Akt and MAPK/Erk pathways that lead to increased proliferation and migration of TNBC cells (Fig. 9). Our study supports that PNPLA8 is a key regulator of TNBC and could be further explored as a potential target for the treatment of TNBC.

### Supplementary Information

**Additional file 1.** Supplementary methods, Tables S1–S6, Figures S1–S12.

## Data Availability

The datasets used and/or analyzed during the current study are available from the corresponding author on reasonable request.

## Abbreviations

AA: Arachidonic acid
CL: Cardiolipin
COX: Cyclooxygenase
CYP: Cytochrome P450
DHA: Docosahexaenoic acid
EETs: Epoxyeicosatrienoic acids
EGFR: Epidermal growth factor receptor
EMT: Epithelial-mesenchymal transition
ER: Estrogen receptor
EP: Prostaglandin E2 receptors
GSEA: Gene Set Enrichment Analysis
GPC: Glycerophosphocholine
GPCR: G protein-coupled receptors
HER2: Human epidermal receptor 2
HETEs: Hydroxyeicosatetraenoic acids
HPETEs: Hydroperoxyeicosatetraenoic acids
LC–MS/MS: Liquid chromatography-tandem mass spectrometry
LT: Leukotriene
LOX: Lipoxygenase
LPC: Lysophosphatidylcholine
MALDI-MS: Matrix-assisted laser desorption/ionization-mass spectrometry
LPCAT: Lysophosphatidylcholine acyltransferases
MAPK: Mitogen-activated protein kinase
PNPLA8: Patatin-like phospholipase domain-containing lipase 8
PA: Phosphatidic acid
PC: Phosphatidylcholine
PE: Phosphatidylethanolamine
PG: Phosphatidylglycerol
PI: Phosphatidylinositol
PI3K: Phosphatidylinositol-3-kinase
PL: Phospholipids
PLA1: Phospholipase A1
PLA2: Phospholipase A2
PLB: Phospholipase B
PLC: Phospholipase C
PLD: Phospholipase D
PR: Progesterone receptor
PS: Phosphatidylserine
ROS: Reactive oxygen species
RTKs: Receptor tyrosine kinases
SM: Sphingomyelin
TCGA: The Cancer Genome Atlas
TNBC: Triple-negative breast cancer
TXB2: Thromboxane 2
TXs: Thromboxanes
