# Supplementary material for: Key regulator PNPLA8 drives phospholipid reprogramming induced proliferation and migration in triple-negative breast cancer
Source: Breast Cancer Res. 2023 Nov 28;25:148. doi: 10.1186/s13058-023-01742-0 (PMC10683240; doi:10.1186/s13058-023-01742-0)
Supplement: Supplementary file 1 — Additional file 1. Supplementary methods, Tables S1–S6, Figures S1–S12. [file 13058_2023_1742_MOESM1_ESM.pdf]

Supplemental Materials

Key regulator PNPLA8 drives phospholipid reprogramming induced proliferation and migration in triple-negative breast cancer

Zheqiong Tan[1,2], Pragney Deme[3], Keerti Boyapati[1], Britt S.R. Claes[4], Annet A.M. Duivenvoorden[5], Ron M.A. Heeren[4], Caitlin M. Tressler [1], Norman James Haughey[3,6], Kristine Glunde[1,7,8,*]

[1]Russell H. Morgan Department of Radiology and Radiological Science, Division of Cancer Imaging Research, Johns Hopkins University School of Medicine, Baltimore, MD, USA

[2]Department of Medical Laboratory, The Central Hospital of Wuhan, Tongji Medical College, Huazhong University of Science and Technology, Wuhan, Hubei, China

[3]Department of Neurology, Johns Hopkins University School of Medicine, Baltimore, MD, USA

[4]Maastricht MultiModal Molecular Imaging Institute, Maastricht University, Maastricht, The Netherlands

[5]Department of Surgery, NUTRIM School of Nutrition and Translational Research in Metabolism, Maastricht University, Maastricht, The Netherlands

[6]Department of Psychiatry, Johns Hopkins University School of Medicine, Baltimore, MD, USA

[7]Sidney Kimmel Comprehensive Cancer Center, Johns Hopkins University School of Medicine, Baltimore, MD, USA

[8]Department of Biological Chemistry, Johns Hopkins University School of Medicine, Baltimore, MD, USA

*Corresponding author. Email: kglunde@mri.jhu.edu Phone: (410) 614-2705 Fax: (410) 614-1948

Supplemental methods

Details of LC-MS/MS lipidomic analysis

In brief, cell homogenates (normalized to their protein amount) gently mixed in a glass tube with ddH2O to make a total volume of 1 mL cell suspension. 2.9 mL of methanol/dichloromethane (2:0.9, v/v) extraction solvent containing twelve internal standards were added to each tube containing the sample and gently mixed. The twelve internal standards were as follows: Cer d18:1/12:0 at 6 ng/mL, SM d18:1/12:0 at 0.3 ng/mL, GlcCer d18:1/12:0 at 3.3 ng/mL, LacCer 18:1/12:0 at 10.6 ng/mL, d5-DAG d16:0/16:0 at 12.5 ng/mL, d5-TAG 16:0/18:0/16:0 at 0.5 ng/mL, cholesteryl-d7 ester 16:0 at 30 ng/mL, PA d12:0/12:0 at 1025 ng/mL, PC 12:0/12:0 at 0.2 ng/mL, PE d12:0/12:0 at 1.6 ng/mL, PG d12:0/12:0 at 200 ng/mL, PS d12:0/12:0 at 900ng/mL. To obtain a biphasic mixture, an additional 1mL of ddH2O, followed by 900µL dichloromethane was added and gently vortexed. The resulting mixture was incubated on ice for 30 min and centrifuged (10 min, 3000g, 4 ºC) to separate organic and aqueous phases. The organic phase containing lipid extracts was collected and stored at -20 ºC. Just prior to analysis, an 800 µL aliquot of the organic layer was dried using a nitrogen evaporator (Organomation Associates, Inc., Berlin, MA, USA) and re-suspended in 150 µl of running solvent (dichloromethane:methanol (1:1) containing 5 mM ammonium acetate), and 5 mg/mL of ceramide C17:0, used to track instrument performance. Lipid analysis was conducted in MS/MS^{ALL} electrospray ion positive mode on a TripleTOF 5600 (AB Sci-ex, Redwood City, CA) quadrupole-time of-flight mass spectrometer (Q-TOF) coupled to a high-performance liquid chromatograph (Shimazu, Canby, OR). Samples (50 µL injection volume) were directly infused by HPLC at a constant flow rate of 7 µL/min using a LC-20AD pump and SIL-20AC XR autosampler. The mass spectrometer was operated at a mass resolution of 30,000 for time of flight (TOF) MS scan and 15,000 for product ion scan (MS/MS) in high sensitivity mode, and the instrument was automatically calibrated after every ten-sample injection using an APCI positive calibration solution delivered through an automatic calibration delivery system (AB SCIEX). The mass spectrometry method details were previously published [45]. Briefly, source parameters were optimized and set as follows: ion source gases at 15 (GSI) and 20 psi (GS2), curtain gas at 30 psi, temperature at 150 °C, positive ion spray voltage at 5,500 V, declustering potential at 80 V, MS scan collision energy set at 10V, and MS/MS scan collision energy set at 40V. LipidView (version 1.3, AB SCIEX, Concord, Ontario, Canada) database was used for the identification and annotation of lipid species from pooled samples. Pooled samples consisting of small aliquots from each experimental group were analyzed to create a targeted list of lipids that were highly reliable and reproducible with a coefficient of variation (CV) of less than 20% (n=8) and detected in seven analytical runs from a total of eight sequential runs. These pre-validated targeted lists of lipids were used to identify unknown lipid features from each individual experimental sample using a targeted processing method by MultiQuant software (version 3.0, AB SCIEX, Concord, Ontario, Canada). For relative quantification, lipid peak intensities were normalized to their corresponding internal standards. Each sample was run in duplicate, and averaged normalized intensities of each lipid were used for statistical analysis. Missing lipid species in a few samples (less than 30% to the total number of samples) were replaced with the lowest normalized intensity obtained within the particular class of lipids.

Supplemental Tables

Table S1. Key resources table

Reagent	Source	Cat#	RRIDs
Antibodies			
anti-PNPLA8 (1:1000)	Sigma-Aldrich (St. Louis, USA)	HPA020083	AB_1851849
anti-PLA2G15 (1:500)	Sigma-Aldrich (St. Louis, USA)	HPA041702	AB_10794486
anti-GAPDH (1:3000)	Sigma-Aldrich (St. Louis, USA)	G8795	AB_1078991
anti-PLA2G4C (1:1000)	Abcam (Cambridge, USA)	ab230250	AB_3073812
anti-LPCAT4 (1:500)	ThermoFisher (Waltham, USA)	PA5-50544	AB_2635997
anti-phospho-Akt (Ser473) (1:1000)	Cell Signaling Technologies (Danvers, USA)	4060S	AB_2315049
anti-Akt (1:1000)	Cell Signaling Technologies (Danvers, USA)	4691S	AB_915783
anti-phospho-Gsk3β(1:500)	Cell Signaling Technologies (Danvers, USA)	9363S	AB_331405
anti-Gsk3β (1:1000)	Cell Signaling Technologies (Danvers, USA)	9315S	AB_490890
Anti-Erk 1/2 (1:1000)	Santa Cruz Biotechnology (Dallas, USA)	sc-514302	AB_2571739
Anti-phospho-Erk 1/2 (Thr202/Tyr 204) (1:1000)	Santa Cruz Biotechnology (Dallas, USA)	sc-136521	AB_10856869
anti-rabbit IgG for WB (1:2000)	Cell Signaling Technologies (Danvers, USA)	7074	AB_2099233
anti-mouse IgG for WB (1:2000)	Cell Signaling Technologies (Danvers, USA)	7076	AB_330924
anti-rabbit IgG for IHC (1:100)	Vectorlabs (Burlingame, CA)	BP-9400-50	AB_3073814
anti-mouse IgG for IHC (1:100)	Vectorlabs (Burlingame, CA)	BP-9200-50	AB_3073815
**Chemicals**	**Source**	**Cat#**	
3,3′-diaminobenzidine (DAB)	Dako (Glostrup, Denmark)	K346889	
Entellan	Merck Millipore (Massachusetts, USA)	1.0796	
Crystal violet	Sigma-Aldrich (St. Louis, USA)	61135	
3-(trimethylsilyl)propionic-2,2,3,3,-d4 acid (TSP)	Sigma-Aldrich (St. Louis, USA)	269913	
Eicosanoid standard cocktail	Cayman Chemical (Ann Arbor, MI, USA)	19667	
Methanol	Honeywell Research Chemicals (Muskegon, MI, USA)	67-56-1	
dichloromethane	Honeywell Research Chemicals (Muskegon, MI, USA)	650463-1L	

Ammonium acetate	Sigma-Aldrich (St. Louis, USA)	73594
1-oleoyl(D7)-2-hydroxy-sn-glycero-3-phosphocholine	Avanti Polar Lipids (Alabaster, AL, USA)	791643
N-lauroyl-D-erythro-sphingosine (Cer d18:1/12:0)	Avanti Polar Lipids (Alabaster, AL, USA)	860512
1,3(D5)-dihexadecanoyl-glycerol (D5-DAG d16:0/16:0)	Avanti Polar Lipids (Alabaster, AL, USA)	800816
D-galactosyl-β-1,1' N-lauroyl-D-erythro-sphingosine (GlcCer d18:1/12:0)	Avanti Polar Lipids (Alabaster, AL, USA)	860543
D-lactosyl- N-lauroyl-D-erythro-sphingosine (LacCer 18:1/12:01,3(d5)	Avanti Polar Lipids (Alabaster, AL, USA)	860576
dihexadecanoyl-2-octadecanoyl-glycerol (D-5TAG 16:0/18:0/16:0)	Avanti Polar Lipids (Alabaster, AL, USA)	860902
cholesteryl-D7 palmitate (cholesteryl-D7 ester 16:0)	Avanti Polar Lipids (Alabaster, AL, USA)	700149
1,2-dilauroyl-sn-glycero-3-phosphate (PA d12:0/12:0)	Avanti Polar Lipids (Alabaster, AL, USA)	840635
1,2-dilauroyl-sn-glycero-3-phosphocholine (PC 12:0/12:0)	Avanti Polar Lipids (Alabaster, AL, USA)	850335
1,2-dilauroyl-sn-glycero-3-phosphoethanolamine (PE d12:0/12:0)	Avanti Polar Lipids (Alabaster, AL, USA)	850702
1,2-dilauroyl-sn-glycero-3-phospho-[1-rac-glycerol] (PG d12:0/12:0)	Avanti Polar Lipids (Alabaster, AL, USA)	840435
1,2-dilauroyl-sn-glycero-3-phospho-L-serine (PS d12:0/12:0)	Avanti Polar Lipids (Alabaster, AL, USA)	840038
N-lauroyl-D-erythro-sphingosylphosphorylcholine (SM d18:1/12:0)	Avanti Polar Lipids (Alabaster, AL, USA)	860583
N-heptadecanoyl-D-erythro-sphingosine (C17 Ceramide (d18:1/17:0))	Avanti Polar Lipids (Alabaster, AL, USA)	860517
Mass calibration solutions	AB Sciex (Concord, CA, USA)	4460131<br>4460134
2-propanol (IPA)	Fisher Scientific (Waltham, US)	67-63-0
acetic acid	Fisher Scientific (Waltham, US)	64-19-7
EGF	Sigma-Aldrich (St. Louis, USA)	SRP3027
insulin	Sigma-Aldrich (St. Louis, USA)	I9278
cholera toxin	Sigma-Aldrich (St. Louis, USA)	227036
Hydrocortisone	Sigma-Aldrich (St. Louis, USA)	3867

PGE2	MedChemExpress (New Jersey, USA)	HY-101952
20-HETE	Merck Millipore (Massachusetts, USA)	H3023
**Critical commercial assays**	**Source**	**Cat#**
RNeasy Mini Kit	QIAGEN (Hilden, Germany)	74104
iScript cDNA Synthesis Kit	Bio-Rad (Hercules, US)	1708890EDU
IQ SYBR Green Supermix	Bio-Rad (Hercules, US)	1708880B10
BCA Protein Assay Kit	Fisher Scientific (Waltham, US)	A55865
ECL Plus Western Blotting Substrate	Fisher Scientific (Waltham, US)	32132X3
MitoSOX Mitochondrial superoxide indicator	Fisher Scientific (Waltham, US)	M36005
Lipofectamine™ 2000	Fisher Scientific (Waltham, US)	11668019
Prostaglandin E2 ELISA Kit	Abcam (Cambridge, USA)	ab133055
DCFDA-Cellular ROS Assay Kit	Abcam (Cambridge, USA)	ab113851
WST-1	Roche (Basel, Switzerland)	5015944001

Table S2. Primer sequences

Gene name	Forward primer sequence	Reverse primer sequence
LYPLA1	CCTTTGCAGGTATCAGAAGTTCA	GCTGCCTGTTTAATCCCAGAT
LYPLA2	CCCTCACGTCAAGTACATCTGT	GACGATTCGATTGGCAGGGAT
LYPLAL1	CCAATGACTGCCCAGAACACCT	TGCCATGCATCCTCCCATAGAG
PLB1	TCCAGCCGTTTCTGAGGACCTA	CAGCCACAACTTGGATGTCTGC
PNPLA6	TCCTCCCTCGTGGATACCTC	GTGGGCGTCTCTTTCTGGA
PNPLA8	GCCCTAATTGGCTATGTGGATCC	TTCGTAGGGTCTGGAGAGCAAC
PLA2G4C	TGCCGGAGTCTCATTTGTCC	GGGTGAACTCGAACCAGGTC
PLA2G15	CAGCGTGGGTTCCTATTTCCA	CCGTTTTCATTTGGGGCTCG
ENPP6	CAGCGATGCTCTTGACTCCTTC	GTCTACAGCCTTGAGGGCATCT
LPCAT1	CGACCTATTCCGAGCCATTGAC	GTGAGGTCTCTGCACAGCTTTC
LPCAT2	GATGGCAGCATTGACTTCCGAG	CCTCCGTTATGTAGCCATCCTC
LPCAT3	GGAGACCTACCTCATCCACCT	CGGCCCATTAGTCGAAGGA
LPCAT4	TCTCGCCTCCAGAGGGTTAAG	AAGAGGACGATAAAGGCCAGA
ABHD3	CCCTGCTTAGACCTTTCATCAC	GAGGCCAGGCAACAATAAGATAG
ACTB	CATGTACGTTGCTATCCAGGC	CTCCTTAATGTCACGCACGAT

Table S3. Dysregulated lipids in TNBC cells compared to controls as determined by volcano plot.

Lipids	p value	Fold change (TNBC/Control)	Log2(TNBC/Control)	-Log10(p)
**Upregulated**				
DAG 40:4	0.02	25.10	4.65	1.68
CE 26:5	0.02	14.74	3.88	1.82
PE O-40:0	0.00	13.62	3.77	15.07
PE O-42:2	0.00	11.53	3.53	9.24
CE 24:6	0.01	11.48	3.52	1.99
DAG 36:0	0.00	10.48	3.39	2.95
CE 26:6	0.01	7.86	2.97	2.28
PC O-40:0	0.00	7.79	2.96	7.10
PE 40:6	0.00	7.62	2.93	10.04
PE O-40:4	0.00	7.33	2.87	9.19
PE O-40:5	0.00	7.15	2.84	5.33
PC O-40:6	0.00	6.45	2.69	3.55
PC O-40:5	0.00	5.60	2.48	2.92
SM 40:0;4	0.00	5.41	2.43	3.45
PC 40:6	0.00	5.07	2.34	4.48
PC 40:5	0.00	4.49	2.17	3.07
TAG 56:7	0.01	4.49	2.17	2.27
PE 40:5	0.00	4.48	2.16	6.78
PC 38:6	0.00	4.48	2.16	8.34
SM 38:0;4	0.00	4.17	2.06	6.49
PC O-38:5	0.00	3.76	1.91	5.11
PC O-42:0	0.00	3.74	1.90	3.55
PC 38:5	0.00	3.66	1.87	5.30
SM 44:4;2	0.00	3.57	1.84	3.12
PC O-40:4	0.00	3.52	1.82	2.63
PC 42:6	0.00	3.51	1.81	3.21
PE 40:4	0.00	3.48	1.80	3.86
PC O-38:6	0.00	3.46	1.79	6.23
PE O-40:1	0.00	3.21	1.68	10.23
PC 38:0	0.00	3.06	1.62	4.01
PE 38:4	0.00	2.87	1.52	7.06
PE O-44:4	0.00	2.83	1.50	5.42
PC O-38:4	0.01	2.83	1.50	1.98
PC 40:0	0.00	2.78	1.48	2.97
PC 40:4	0.01	2.71	1.44	2.01
TAG 46:6	0.00	2.68	1.42	3.63
SM 40:4;3	0.02	2.60	1.38	1.63
SM 42:4;2	0.00	2.56	1.36	4.20
SM 42:0;4	0.00	2.42	1.27	5.27
PC 42:0	0.02	2.34	1.23	1.73
PS 38:4	0.00	2.23	1.16	3.78

PS 38:3	0.01	2.21	1.14	2.06
SM 42:1;4	0.00	2.21	1.14	2.80
Hex2Cer 34:1;3	0.00	2.20	1.14	2.61
PC O-46:5	0.03	2.19	1.13	1.48
PC O-36:5	0.00	2.17	1.12	7.41
PC O-42:4	0.01	2.13	1.09	2.00
PE 38:5	0.00	2.02	1.01	4.76
**Downregulated**				
SM 40:2;2	0.00	0.49	-1.02	9.43
PG 36:5	0.00	0.49	-1.03	7.34
TAG 54:10	0.00	0.49	-1.03	3.04
PC O-34:2	0.00	0.49	-1.03	10.53
PC 34:3	0.00	0.49	-1.03	12.97
PE 38:1	0.04	0.47	-1.09	1.45
PC O-36:3	0.00	0.45	-1.17	11.23
TAG 54:4	0.00	0.42	-1.24	2.96
SM 36:2;3	0.00	0.40	-1.31	15.37
LPC 18:0	0.00	0.36	-1.48	6.01
TAG 46:4	0.00	0.31	-1.71	4.55
LPC 18:2	0.00	0.26	-1.97	7.10
PC O-34:3	0.00	0.25	-2.00	16.05
PE 36:3	0.00	0.19	-2.37	19.50
TAG 42:3	0.00	0.03	-5.08	3.69
TAG 44:6	0.00	0.02	-5.38	3.60

Table S4. Dysregulated lipids in TNBC cells compared to controls as determined by s-plot.

Lipids	M2.p[1]	M2.p(corr)[1]
**Upregulated**		
PE O-40:0	0.19	0.90
PE O-42:2	0.17	0.89
PC O-40:0	0.14	0.89
PE 40:6	0.15	0.88
SM 38:0;4	0.12	0.86
PC 38:6	0.13	0.83
PC O-38:6	0.11	0.83
DAG 36:0	0.13	0.80
PC 38:5	0.11	0.79
PC 40:6	0.13	0.79
PC O-38:5	0.10	0.78
PE O-40:1	0.12	0.77
PE O-40:4	0.14	0.77
PE 38:4	0.10	0.77
PE O-40:5	0.15	0.77
PC O-36:5	0.09	0.77
SM 42:0;4	0.08	0.75
PE 40:5	0.13	0.74
PC O-40:6	0.11	0.73
PC O-42:0	0.10	0.73
SM 42:4;2	0.09	0.73
SM 40:0;4	0.11	0.72
PE 40:4	0.11	0.71
PC 40:5	0.11	0.71
**Downregulated**		
PC O-36:3	-0.08	-0.71
LPC 18:2	-0.12	-0.72
PG O-38:0	-0.07	-0.73
PC O-34:2	-0.08	-0.74
PS 36:5	-0.07	-0.77
SM 38:2;2	-0.07	-0.82
SM 36:2;3	-0.09	-0.83
PC 34:3	-0.08	-0.86
PE 36:3	-0.13	-0.87
PC O-34:3	-0.12	-0.93

Table S5. Dysregulated lipids in Hs578T-siPNPLA8 compared to controls.

Lipids	Fold change	*p* value
**Upregulated**		
PG 38:3	1184.70	0.04
Hex2Cer 30:1;3	76.78	0.00
LPC 20:0	1.81	0.00
PC 40:1	1.57	0.00
PG O-38:0	1.54	0.01
PC 42:0	1.53	0.00
PG 36:5	1.51	0.04
Hex2Cer 34:1;2	1.45	0.00
PC O-36:0	1.44	0.00
PC 38:1	1.41	0.00
PC O-32:0	1.40	0.00
LPC 30:0	1.40	0.00
PC O-36:6	1.40	0.00
PC 38:2	1.37	0.00
LPC 18:0	1.36	0.00
LPC 16:0	1.36	0.05
PC 44:1	1.35	0.00
PC O-38:3	1.34	0.00
PC 44:0	1.33	0.00
PC 34:2	1.28	0.00
PC 34:0	1.28	0.00
PC O-40:3	1.27	0.03
PC O-34:0	1.26	0.01
PC 36:1	1.26	0.00
PC O-34:2	1.26	0.01
PC 36:2	1.26	0.00
PC O-34:1	1.25	0.01
PC O-36:3	1.25	0.00
PC O-44:6	1.25	0.02
PC 40:3	1.24	0.03
PC O-36:1	1.24	0.00
Hex2Cer 30:1;2	1.24	0.03
PC 40:0	1.24	0.00
PC O-38:2	1.24	0.00
PC O-36:2	1.22	0.01
PC 38:3	1.22	0.01
PC 38:0	1.22	0.01
PC 36:0	1.21	0.01
PC 36:3	1.21	0.02
LPC O-30:0	1.20	0.02

LPC 30:1	1.20	0.00
PC O-34:3	1.20	0.00
PC 34:1	1.19	0.00
PC 32:1	1.19	0.00
PC 26:1	1.18	0.03
PC 28:0	1.18	0.01
PC 30:1	1.17	0.01
PC O-46:5	1.15	0.05
PC 30:0	1.12	0.03
PC 32:0	1.12	0.01
PC O-42:4	1.10	0.05
PC 24:0	1.09	0.02
**Downregulated**		
HexCer 44:0;4	0.01	0.00
HexCer 44:1;4	0.02	0.00
HexCer 46:1;4	0.02	0.00
HexCer 38:1;2	0.71	0.01
HexCer 40:1;2	0.80	0.02
HexCer 42:1;2	0.90	0.03

Table S6. Dysregulated lipids in SUM159PT-siPNPLA8 compared to controls.

Lipids	Fold change	*p* value
**Upregulated**		
Hex2Cer 34:1;2	1.30	0.02
LPC 16:0	1.29	0.00
PG O-38:0	1.27	0.01
PC 42:0	1.27	0.00
PC O-34:6	1.23	0.02
PG 36:5	1.21	0.03
LPC 18:0	1.18	0.01
PC 44:1	1.18	0.00
PC O-40:0	1.17	0.02
PC 40:0	1.17	0.00
PC 44:0	1.13	0.00
PC 38:0	1.13	0.03
PC 40:1	1.10	0.00
PC O-42:0	1.10	0.01
PC 38:6	1.10	0.01
PC 34:0	1.09	0.00
PC 28:0	1.08	0.03
PC 38:1	1.07	0.01
PC O-32:0	1.05	0.01
**Downregulated**		
HexCer 46:1;4	0.01	0.00
HexCer 44:1;4	0.02	0.00
HexCer 38:2;3	0.02	0.00
HexCer 38:1;2	0.57	0.00
HexCer 40:2;2	0.75	0.04
HexCer 40:1;2	0.81	0.01
HexCer 42:2;2	0.86	0.00
HexCer 42:1;2	0.89	0.00

Supplemental Figures

Figure S1

A

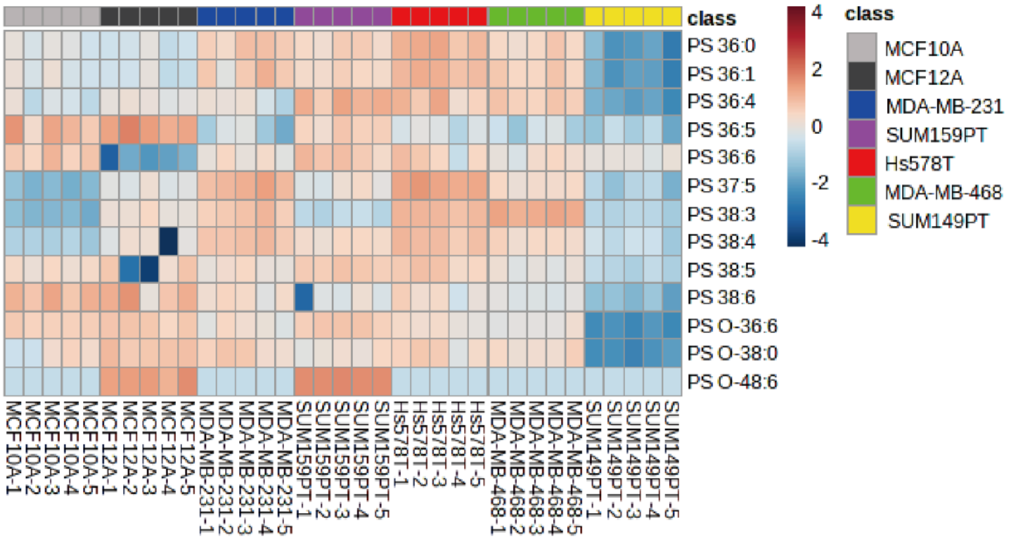

B

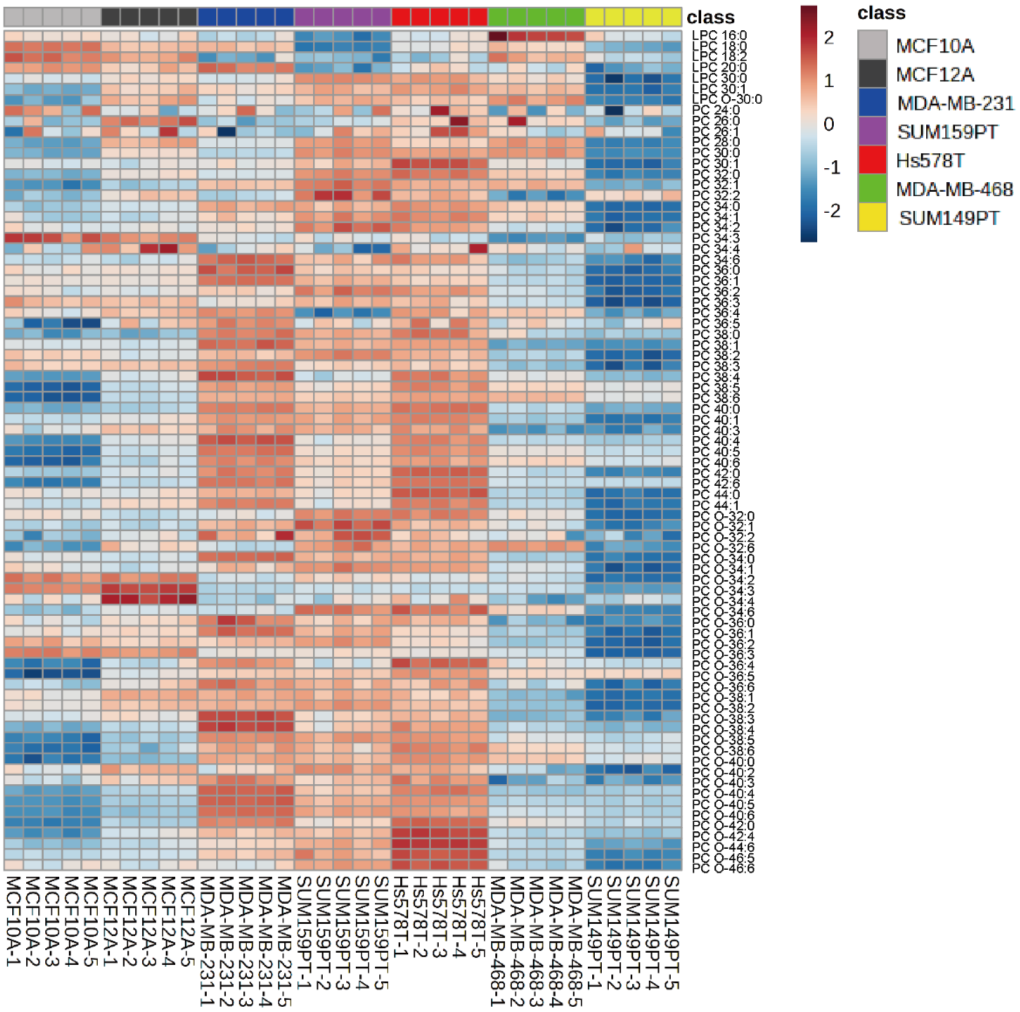

C

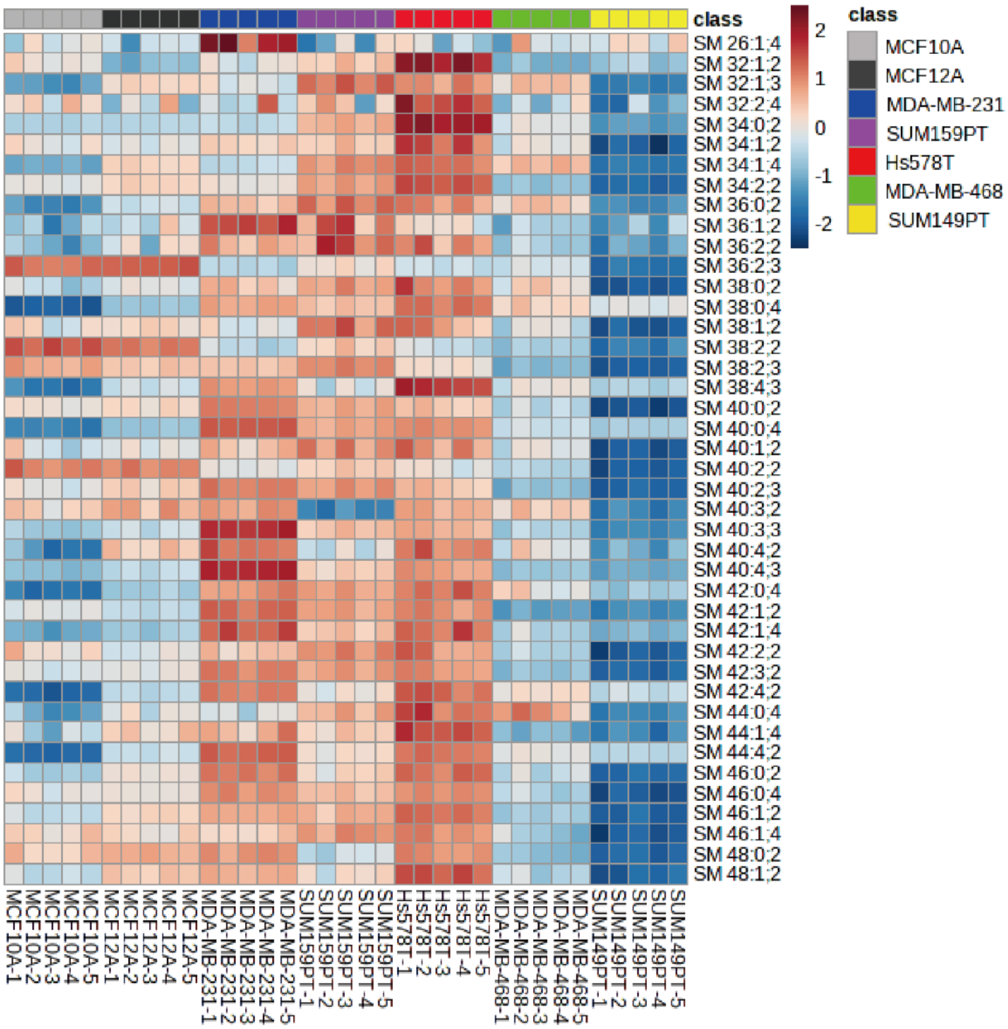

class
SM 26:1;4
SM 32:1;2
SM 32:1;3
SM 32:2;4
SM 34:0;2
SM 34:1;2
SM 34:1;4
SM 34:2;2
SM 36:0;2
SM 36:1;2
SM 36:2;2
SM 36:2;3
SM 38:0;2
SM 38:0;4
SM 38:1;2
SM 38:2;2
SM 38:2;3
SM 38:4;3
SM 40:0;2
SM 40:0;4
SM 40:1;2
SM 40:2;2
SM 40:2;3
SM 40:3;2
SM 40:3;3
SM 40:4;2
SM 40:4;3
SM 42:0;4
SM 42:1;2
SM 42:1;4
SM 42:2;2
SM 42:3;2
SM 42:4;2
SM 44:0;4
SM 44:1;4
SM 44:4;2
SM 46:0;2
SM 46:0;4
SM 46:1;2
SM 46:1;4
SM 48:0;2
SM 48:1;2
2
1
0
-1
-2
class
MCF10A
MCF12A
MDA-MB-231
SUM159PT
Hs578T
MDA-MB-468
SUM149PT
MCF10A-1
MCF10A-2
MCF10A-3
MCF10A-4
MCF10A-5
MCF12A-1
MCF12A-2
MCF12A-3
MCF12A-4
MCF12A-5
MDA-MB-231-1
MDA-MB-231-2
MDA-MB-231-3
MDA-MB-231-4
MDA-MB-231-5
SUM159PT-1
SUM159PT-2
SUM159PT-3
SUM159PT-4
SUM159PT-5
Hs578T-1
Hs578T-2
Hs578T-3
Hs578T-4
Hs578T-5
MDA-MB-468-1
MDA-MB-468-2
MDA-MB-468-3
MDA-MB-468-4
MDA-MB-468-5
SUM149PT-1
SUM149PT-2
SUM149PT-3
SUM149PT-4
SUM149PT-5

D

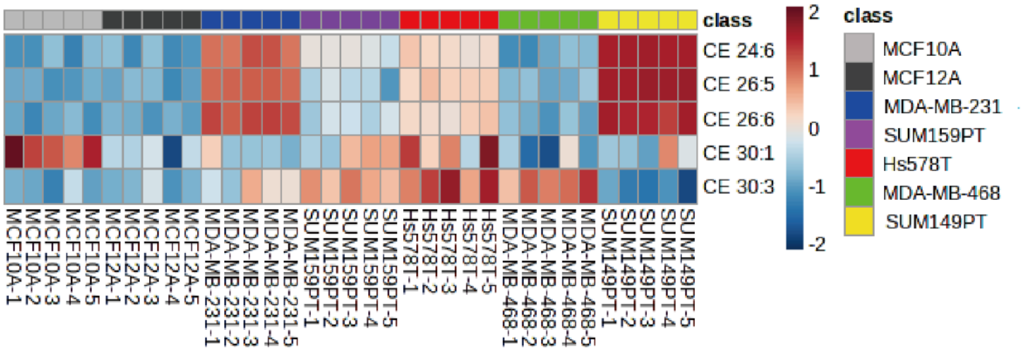

class
CE 24:6
CE 26:5
CE 26:6
CE 30:1
CE 30:3
2
1
0
-1
-2
class
MCF10A
MCF12A
MDA-MB-231
SUM159PT
Hs578T
MDA-MB-468
SUM149PT
MCF10A-1
MCF10A-2
MCF10A-3
MCF10A-4
MCF10A-5
MCF12A-1
MCF12A-2
MCF12A-3
MCF12A-4
MCF12A-5
MDA-MB-231-1
MDA-MB-231-2
MDA-MB-231-3
MDA-MB-231-4
MDA-MB-231-5
SUM159PT-1
SUM159PT-2
SUM159PT-3
SUM159PT-4
SUM159PT-5
Hs578T-1
Hs578T-2
Hs578T-3
Hs578T-4
Hs578T-5
MDA-MB-468-1
MDA-MB-468-2
MDA-MB-468-3
MDA-MB-468-4
MDA-MB-468-5
SUM149PT-1
SUM149PT-2
SUM149PT-3
SUM149PT-4
SUM149PT-5

E

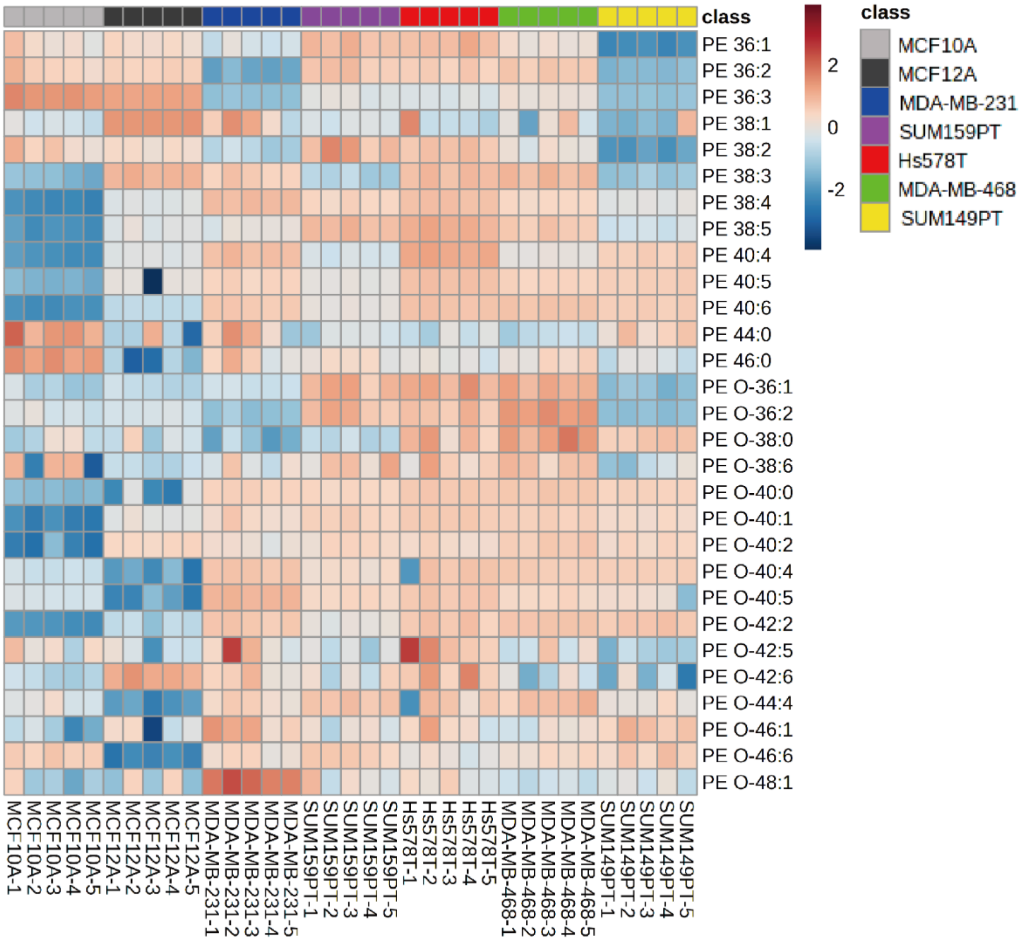
class
PE 36:1
PE 36:2
PE 36:3
PE 38:1
PE 38:2
PE 38:3
PE 38:4
PE 38:5
PE 40:4
PE 40:5
PE 40:6
PE 44:0
PE 46:0
PE O-36:1
PE O-36:2
PE O-38:0
PE O-38:6
PE O-40:0
PE O-40:1
PE O-40:2
PE O-40:4
PE O-40:5
PE O-42:2
PE O-42:5
PE O-42:6
PE O-44:4
PE O-46:1
PE O-46:6
PE O-48:1
MCF10A-1
MCF10A-2
MCF10A-3
MCF10A-4
MCF10A-5
MCF12A-1
MCF12A-2
MCF12A-3
MCF12A-4
MCF12A-5
MDA-MB-231-1
MDA-MB-231-2
MDA-MB-231-3
MDA-MB-231-4
MDA-MB-231-5
SUM159PT-1
SUM159PT-2
SUM159PT-3
SUM159PT-4
SUM159PT-5
Hs578T-1
Hs578T-2
Hs578T-3
Hs578T-4
Hs578T-5
MDA-MB-468-1
MDA-MB-468-2
MDA-MB-468-3
MDA-MB-468-4
MDA-MB-468-5
SUM149PT-1
SUM149PT-2
SUM149PT-3
SUM149PT-4
SUM149PT-5
2
0
-2
class
MCF10A
MCF12A
MDA-MB-231
SUM159PT
Hs578T
MDA-MB-468
SUM149PT

F

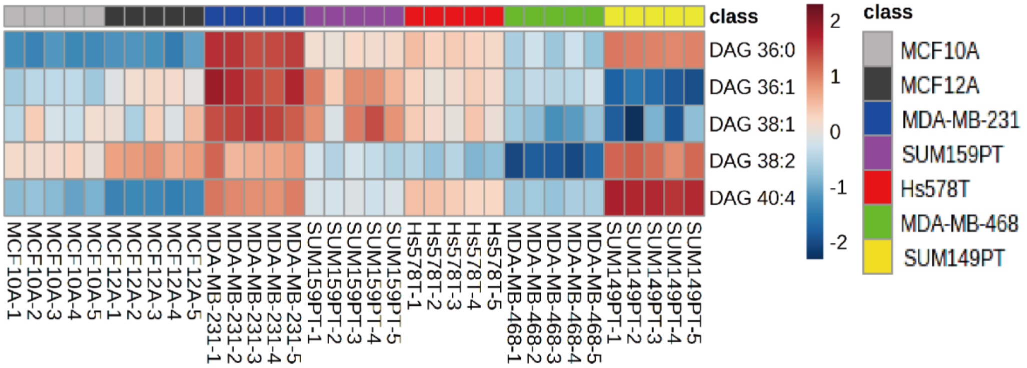
class
DAG 36:0
DAG 36:1
DAG 38:1
DAG 38:2
DAG 40:4
MCF10A-1
MCF10A-2
MCF10A-3
MCF10A-4
MCF10A-5
MCF12A-1
MCF12A-2
MCF12A-3
MCF12A-4
MCF12A-5
MDA-MB-231-1
MDA-MB-231-2
MDA-MB-231-3
MDA-MB-231-4
MDA-MB-231-5
SUM159PT-1
SUM159PT-2
SUM159PT-3
SUM159PT-4
SUM159PT-5
Hs578T-1
Hs578T-2
Hs578T-3
Hs578T-4
Hs578T-5
MDA-MB-468-1
MDA-MB-468-2
MDA-MB-468-3
MDA-MB-468-4
MDA-MB-468-5
SUM149PT-1
SUM149PT-2
SUM149PT-3
SUM149PT-4
SUM149PT-5
2
1
0
-1
-2
class
MCF10A
MCF12A
MDA-MB-231
SUM159PT
Hs578T
MDA-MB-468
SUM149PT

G

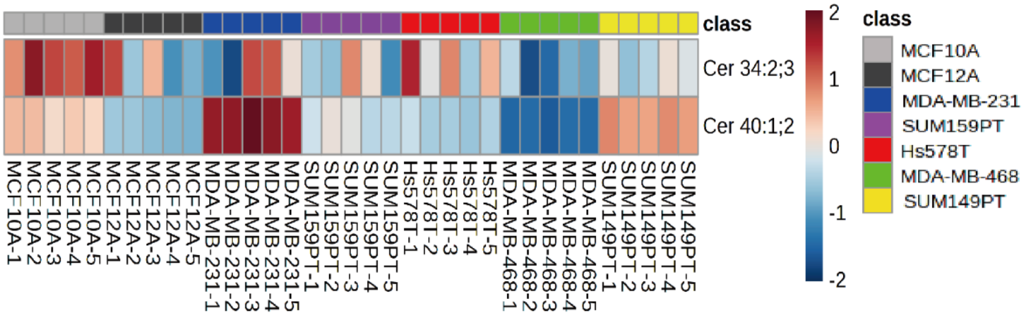
class
Cer 34:2;3
Cer 40:1;2
MCF10A-1
MCF10A-2
MCF10A-3
MCF10A-4
MCF10A-5
MCF12A-1
MCF12A-2
MCF12A-3
MCF12A-4
MCF12A-5
MDA-MB-231-1
MDA-MB-231-2
MDA-MB-231-3
MDA-MB-231-4
MDA-MB-231-5
SUM159PT-1
SUM159PT-2
SUM159PT-3
SUM159PT-4
SUM159PT-5
Hs578T-1
Hs578T-2
Hs578T-3
Hs578T-4
Hs578T-5
MDA-MB-468-1
MDA-MB-468-2
MDA-MB-468-3
MDA-MB-468-4
MDA-MB-468-5
SUM149PT-1
SUM149PT-2
SUM149PT-3
SUM149PT-4
SUM149PT-5
2
1
0
-1
-2
class
MCF10A
MCF12A
MDA-MB-231
SUM159PT
Hs578T
MDA-MB-468
SUM149PT

H

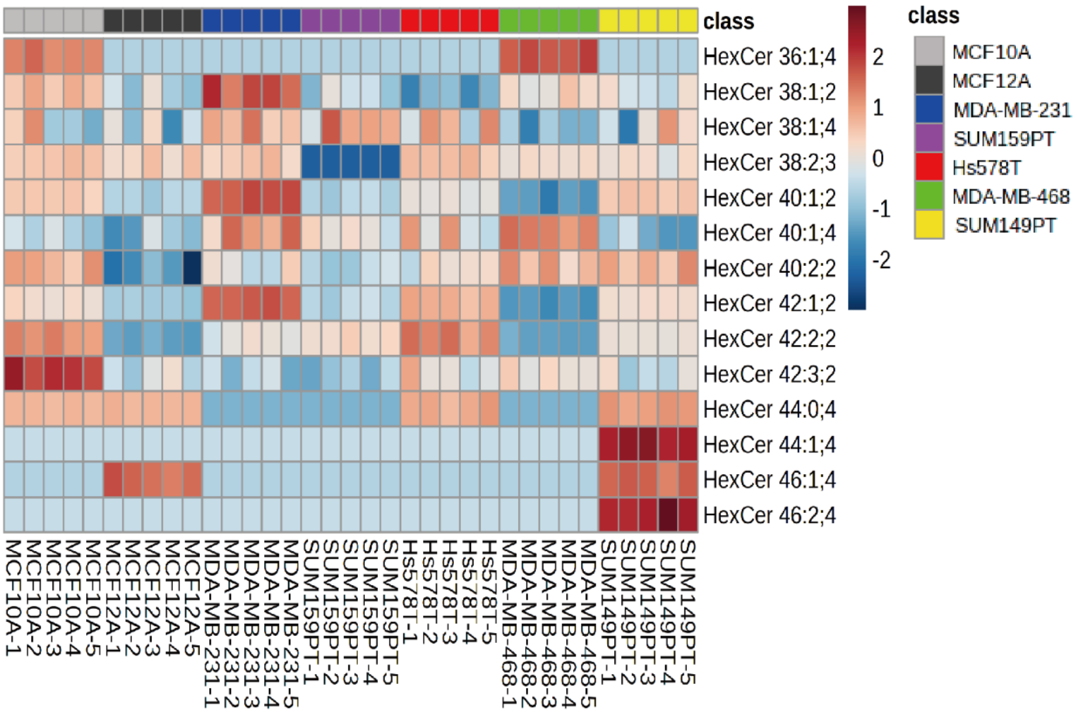
class
HexCer 36:1;4
HexCer 38:1;2
HexCer 38:1;4
HexCer 38:2;3
HexCer 40:1;2
HexCer 40:1;4
HexCer 40:2;2
HexCer 42:1;2
HexCer 42:2;2
HexCer 42:3;2
HexCer 44:0;4
HexCer 44:1;4
HexCer 46:1;4
HexCer 46:2;4
2
1
0
-1
-2
class
MCF10A
MCF12A
MDA-MB-231
SUM159PT
Hs578T
MDA-MB-468
SUM149PT
MCF10A-1
MCF10A-2
MCF10A-3
MCF10A-4
MCF10A-5
MCF12A-1
MCF12A-2
MCF12A-3
MCF12A-4
MCF12A-5
MDA-MB-231-1
MDA-MB-231-2
MDA-MB-231-3
MDA-MB-231-4
MDA-MB-231-5
SUM159PT-1
SUM159PT-2
SUM159PT-3
SUM159PT-4
SUM159PT-5
Hs578T-1
Hs578T-2
Hs578T-3
Hs578T-4
Hs578T-5
MDA-MB-468-1
MDA-MB-468-2
MDA-MB-468-3
MDA-MB-468-4
MDA-MB-468-5
SUM149PT-1
SUM149PT-2
SUM149PT-3
SUM149PT-4
SUM149PT-5

I

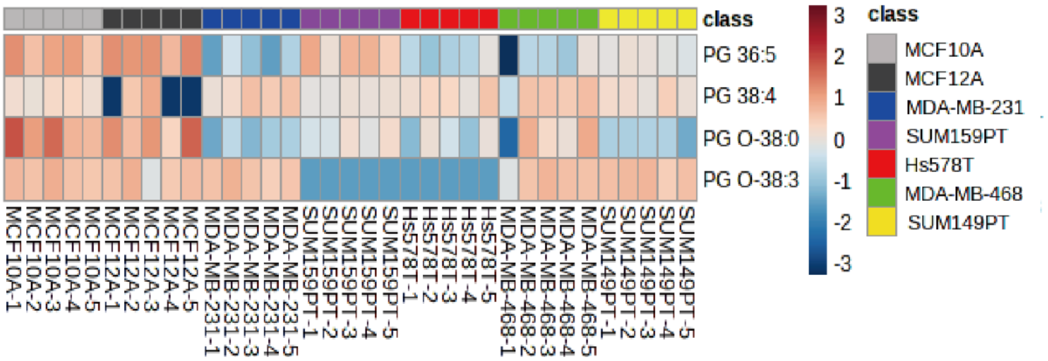
class
PG 36:5
PG 38:4
PG O-38:0
PG O-38:3
3
2
1
0
-1
-2
-3
class
MCF10A
MCF12A
MDA-MB-231
SUM159PT
Hs578T
MDA-MB-468
SUM149PT
MCF10A-1
MCF10A-2
MCF10A-3
MCF10A-4
MCF10A-5
MCF12A-1
MCF12A-2
MCF12A-3
MCF12A-4
MCF12A-5
MDA-MB-231-1
MDA-MB-231-2
MDA-MB-231-3
MDA-MB-231-4
MDA-MB-231-5
SUM159PT-1
SUM159PT-2
SUM159PT-3
SUM159PT-4
SUM159PT-5
Hs578T-1
Hs578T-2
Hs578T-3
Hs578T-4
Hs578T-5
MDA-MB-468-1
MDA-MB-468-2
MDA-MB-468-3
MDA-MB-468-4
MDA-MB-468-5
SUM149PT-1
SUM149PT-2
SUM149PT-3
SUM149PT-4
SUM149PT-5

J

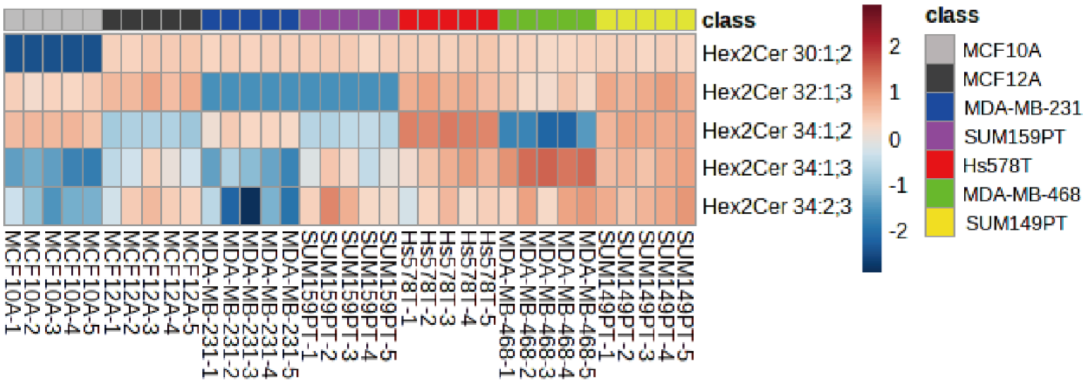
class
Hex2Cer 30:1;2
Hex2Cer 32:1;3
Hex2Cer 34:1;2
Hex2Cer 34:1;3
Hex2Cer 34:2;3
2
1
0
-1
-2
class
MCF10A
MCF12A
MDA-MB-231
SUM159PT
Hs578T
MDA-MB-468
SUM149PT
MCF10A-1
MCF10A-2
MCF10A-3
MCF10A-4
MCF10A-5
MCF12A-1
MCF12A-2
MCF12A-3
MCF12A-4
MCF12A-5
MDA-MB-231-1
MDA-MB-231-2
MDA-MB-231-3
MDA-MB-231-4
MDA-MB-231-5
SUM159PT-1
SUM159PT-2
SUM159PT-3
SUM159PT-4
SUM159PT-5
Hs578T-1
Hs578T-2
Hs578T-3
Hs578T-4
Hs578T-5
MDA-MB-468-1
MDA-MB-468-2
MDA-MB-468-3
MDA-MB-468-4
MDA-MB-468-5
SUM149PT-1
SUM149PT-2
SUM149PT-3
SUM149PT-4
SUM149PT-5

K

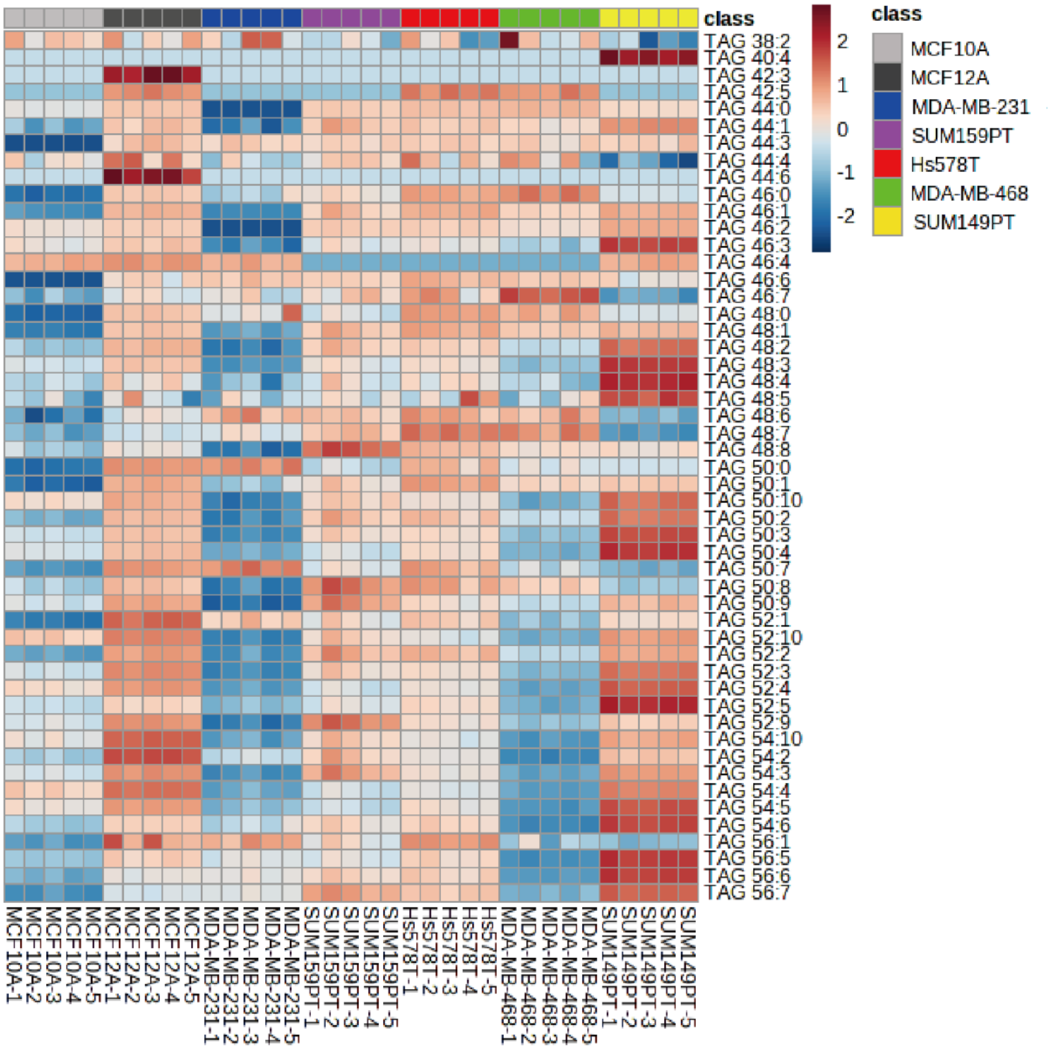

Figure S1. Relative abundance of lipid species in triple-negative breast cancer cell lines. Heatmap of log normalized abundance of lipid species of PS **(A)**, PC **(B)**, SM **(C)**, CE **(D)**, PE **(E)**, DAG **(F)**, Cer **(G)**, HerCer **(H)**, PG **(I)**, Her2Cer **(J)** and TAG **(K)** in TNBC (MDA-MB-231, SUM159PT, Hs578T, MDA-MB-468, SUM149PT) and nonmalignant human mammary epithelial cell lines (MCF10A, MCF12A). Each cell line is represented by 5 biological replicates.

Figure S2

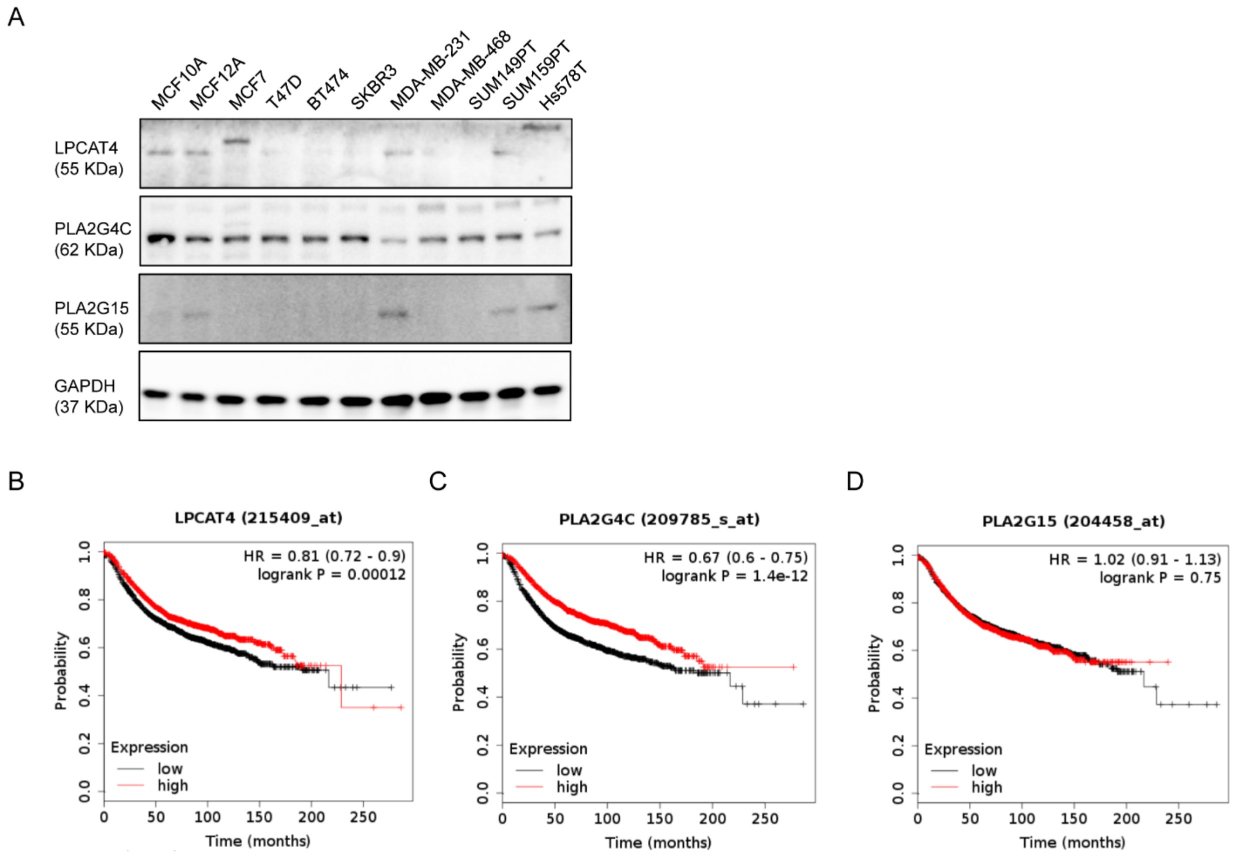

Figure S2. Protein expression levels of PLA2G4C, PLA2G15 and LPCAT4 in breast cancer cell lines and their correlation with breast cancer patient survival. **(A)** Protein expression levels of LPCAT4, PLA2G4C and PLA2G15 in a panel of human mammary epithelial cell lines (MCF10A, MCF12A) and breast cancer cell lines (MCF7, T47D, BT474, SKBR3, MDA-MB-231, MDA-MB-468, SUM149PT, SUM159PT, Hs578T). GAPDH was used as loading control. **(B)** Survival rates of breast cancer patients with low (n = 1995) or high (n =1956) expression levels of LPCAT4. **(C)** Survival rates of breast cancer patients with low (n = 1987) or high (n =1964) expression levels of PLA2G4C. **(D)** Survival rates of breast cancer patients with low (n = 1987) or high (n =1964) expression levels of PLA2G15. The survival curves were obtained by KM-plotter.

Figure S3

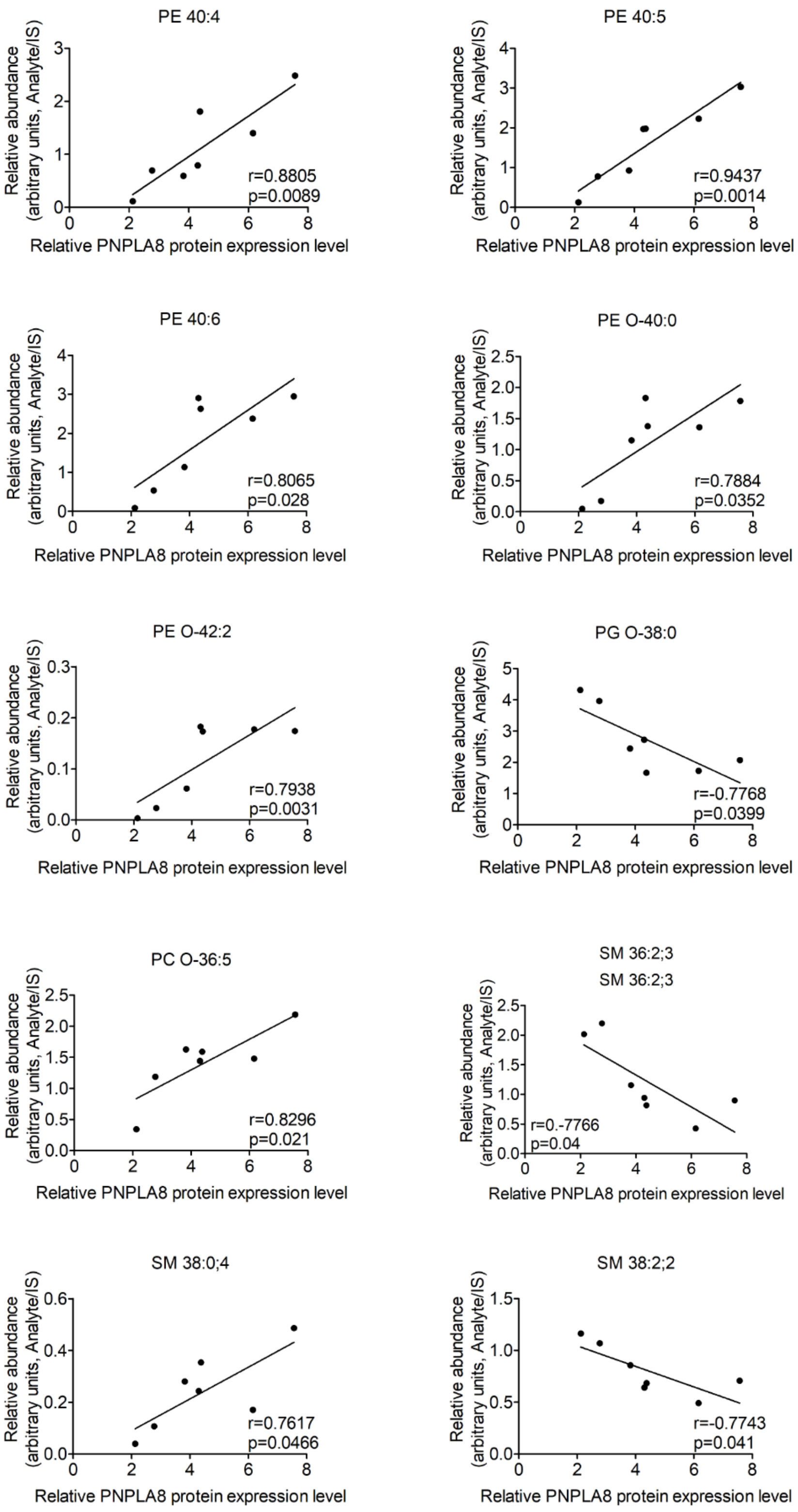

Figure S3. Correlation analysis of different cellular phospholipid species levels with PNPLA8 protein levels. PNPLA8 protein levels were normalized to GAPDH protein levels.

Figure S4

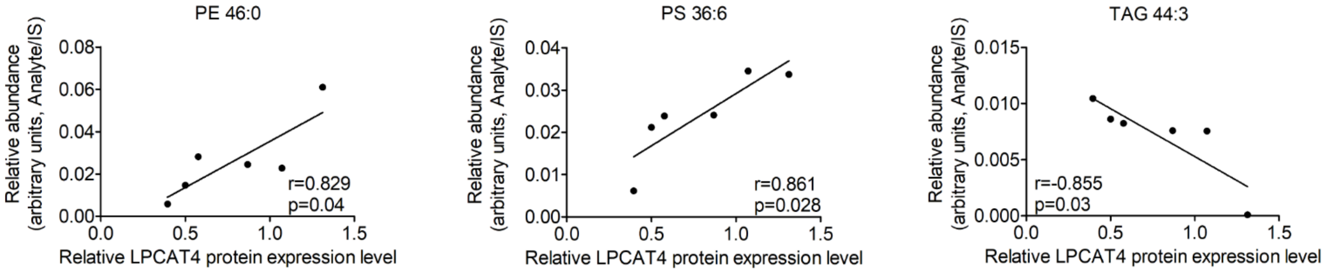

Figure S4. Correlation analysis of different cellular phospholipid species levels with LPCAT4 protein levels. LPCAT4 protein levels were normalized to GAPDH protein levels.

Figure S5

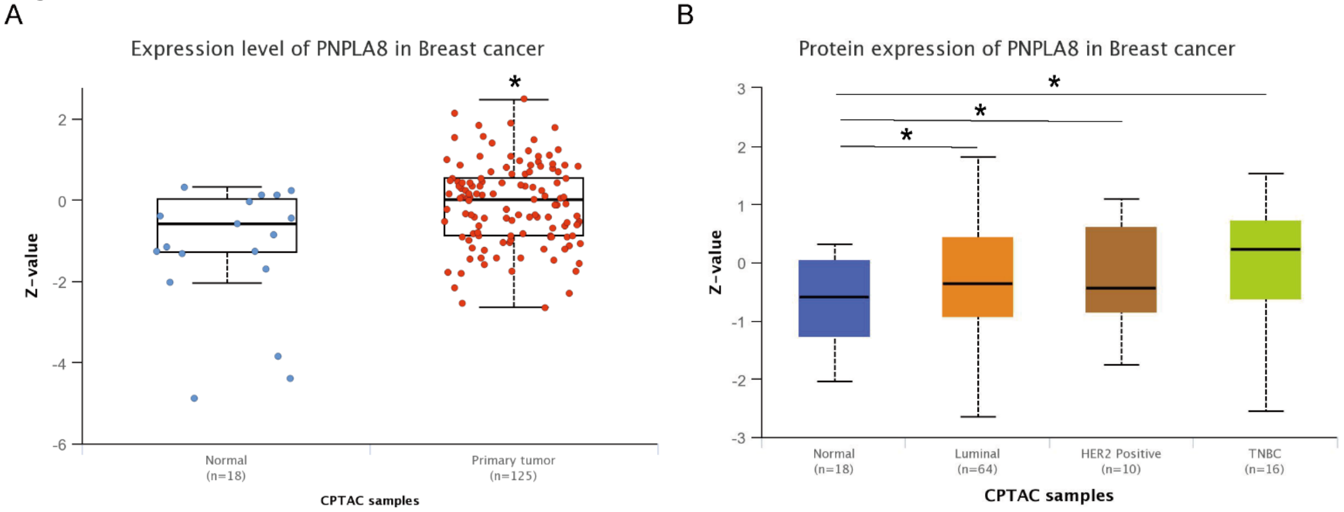

Figure S5. PNPLA8 protein expression levels of breast cancer tissues of CPTAC breast cancer dataset. (A) PNPLA8 protein expression levels of normal breast tissues and primary breast cancer tissues. **(B)** PNPLA8 protein expression levels of normal breast tissues and different molecular subtypes of breast cancer tissues. *$p<0.05$.

Figure S6

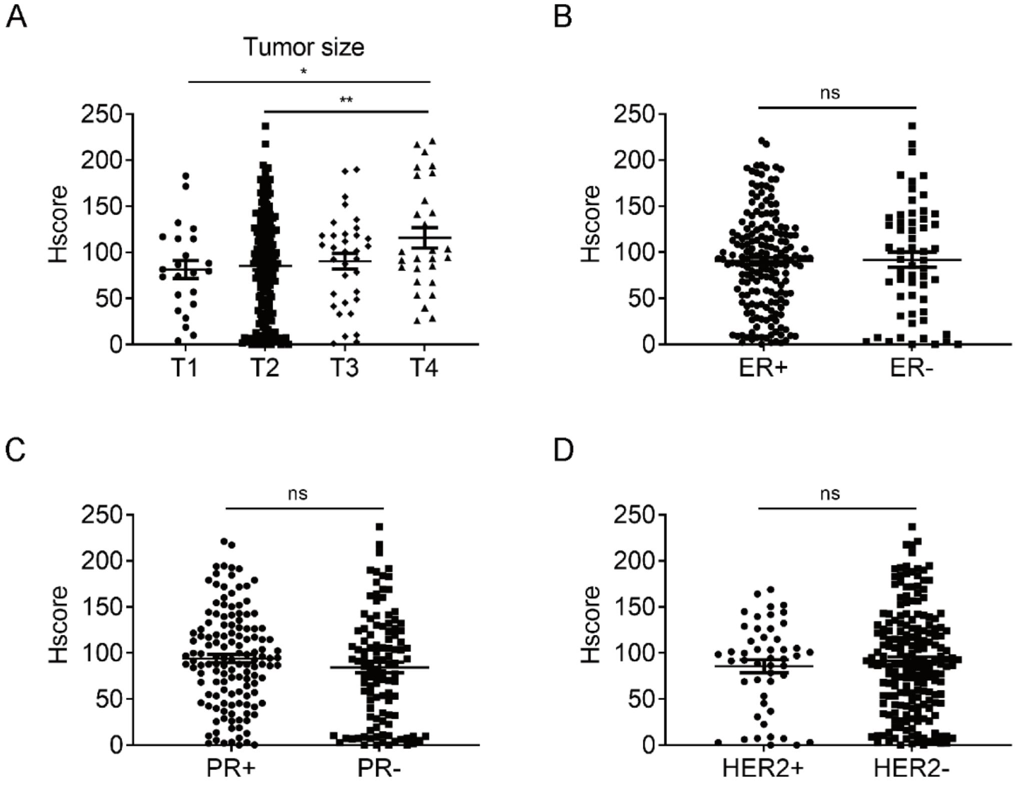

Figure S6. PNPLA8 expression levels in breast cancer tissues with different tumor size, ER, PR and HER2 expression status. (A) PNPLA8 expression levels in breast cancer tissues with T1 (maximum diameter≤20 mm), T2 (> 20 mm, ≤50 mm), T3 (> 50 mm) and T4 (tumor invade thoracicwall or skin) tumor size. **(B-D)** PNPLA8 expression levels in breast cancer tissues with positive or negative expression of ER, PR and HER2. *p<0.05, **p<0.01.

Figure S7

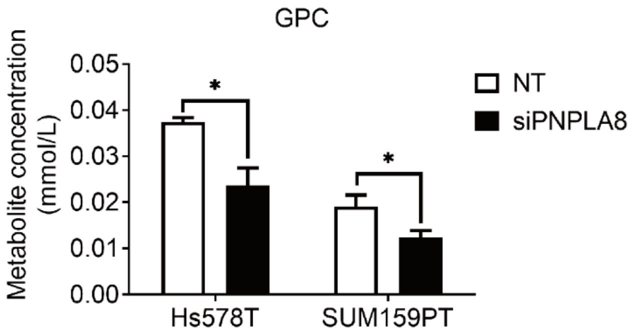

Figure S7. Cellular levels of GPC in Hs578T and SUM159PT cells treated with PNPLA8 siRNA or scrambled siRNA control as measured by high resolution (HR) ^{1}H MRS. *$p<0.05$.

Figure S8

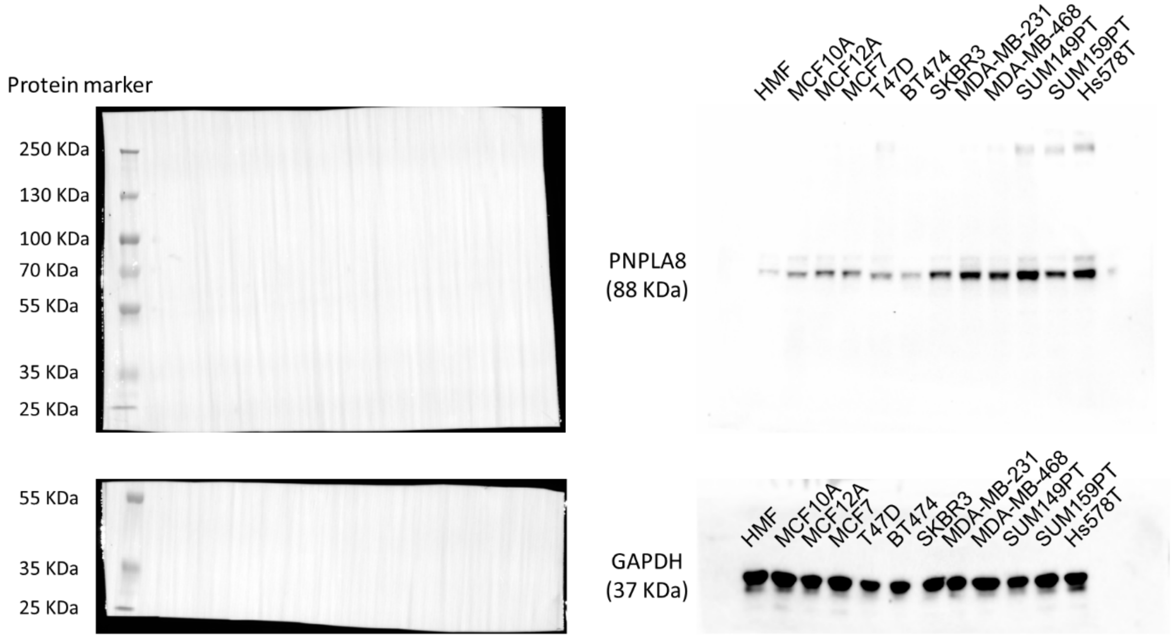

Figure S8. Full uncropped Western Blot images of Figure 3B. Protein expression levels of PNPLA8 and GAPDH in a panel of human mammary fibroblast cell line (HMF), human mammary epithelial cell lines (MCF10A, MCF12A) and breast cancer cell lines (MCF7, T47D, BT474, SKBR3, MDA-MB-231, MDA-MB-468, SUM149PT, SUM159PT, Hs578T). GAPDH was used as loading control.

Figure S9

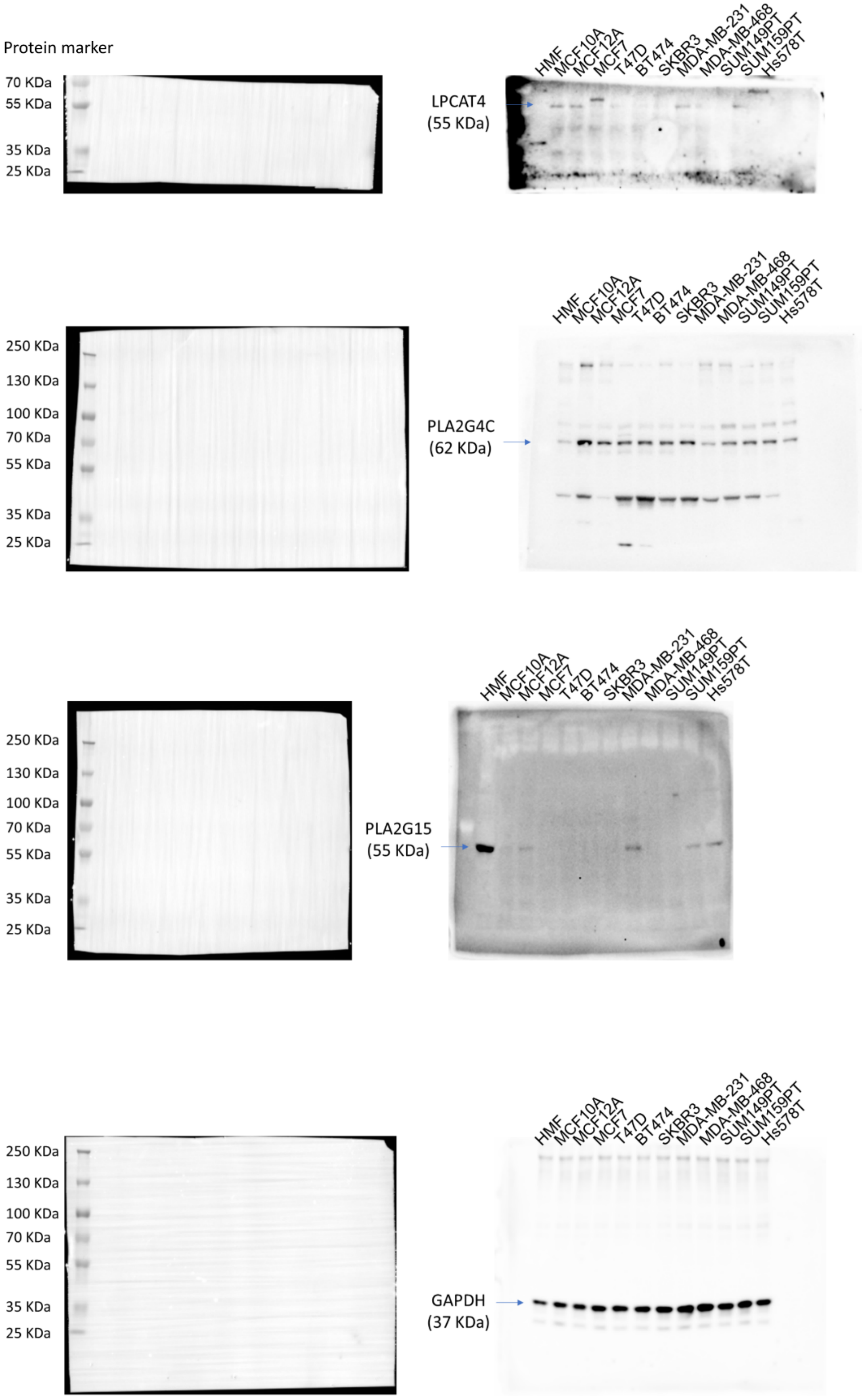

Protein marker
70 KDa
55 KDa
35 KDa
25 KDa
HMF
MCF10A
MCF12A
MCF7
T47D
BT474
SKBR3
MDA-MB-231
MDA-MB-468
SUM149PT
SUM159PT
Hs578T
LPCAT4
(55 KDa)
250 KDa
130 KDa
100 KDa
70 KDa
55 KDa
35 KDa
25 KDa
PLA2G4C
(62 KDa)
PLA2G15
(55 KDa)
GAPDH
(37 KDa)

Figure S9. Full uncropped Western Blot images of Fig. S2A. Protein expression levels of LPACAT4, PLA2G4C, PLA2G15 and GAPDH in a panel of human mammary fibroblast cell line (HMF), human mammary epithelial cell lines (MCF10A, MCF12A) and breast cancer cell lines (MCF7, T47D, BT474, SKBR3, MDA-MB-231, MDA-MB-468, SUM149PT, SUM159PT, Hs578T). GAPDH was used as loading control.

Figure S10

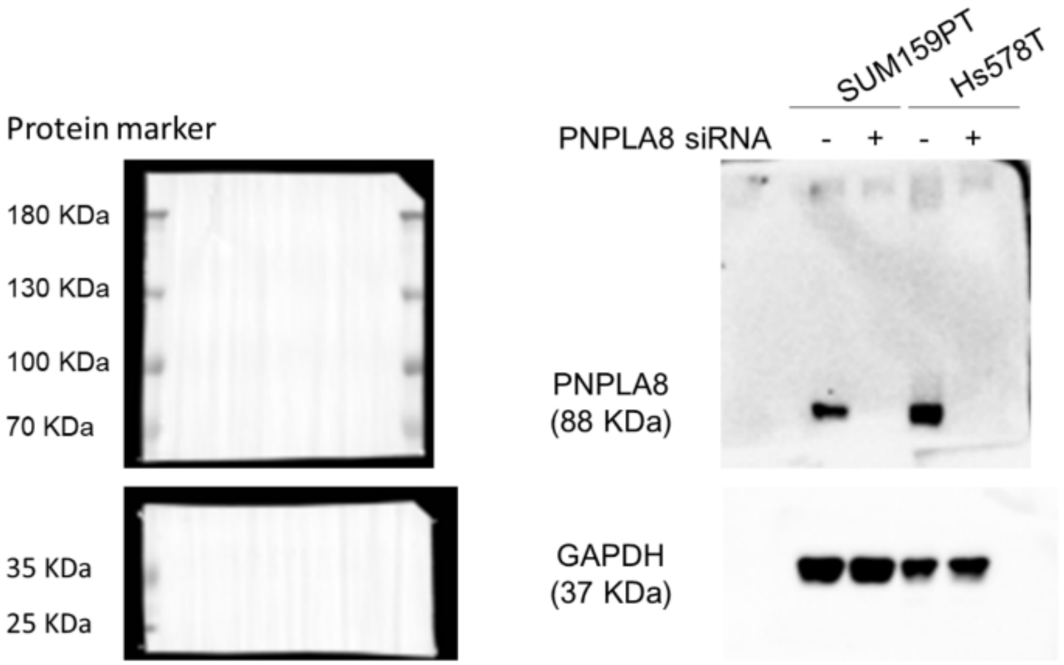

Figure S10. Full uncropped Western Blot images of Fig. 5B. Protein expression level of PNPLA8 and GAPDH in SUM159PT and Hs578T cell lines treated with PNPLA8 siRNA or scrambled siRNA. GAPDH was used as loading control.

Figure S11

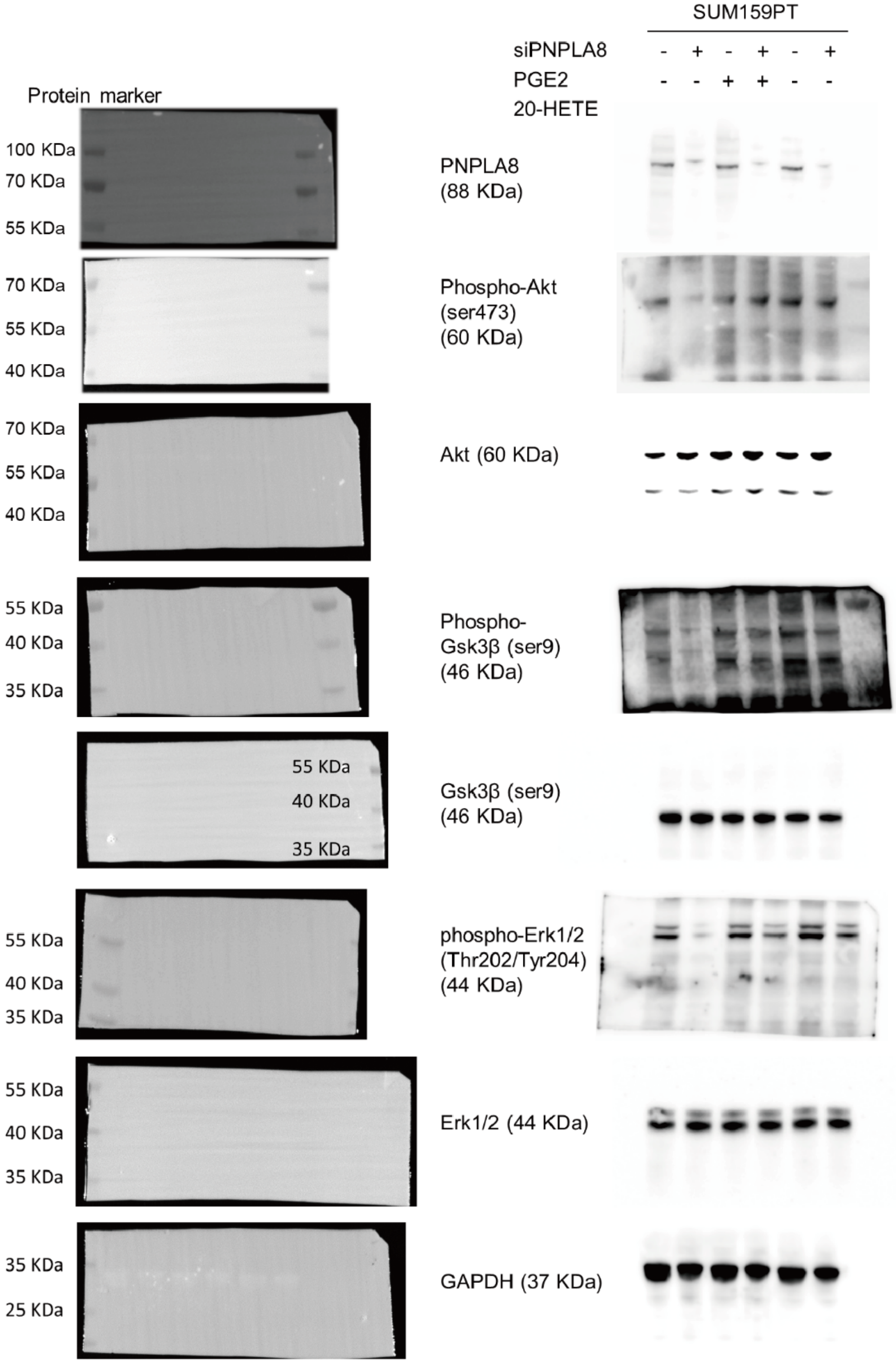

Figure S11. Full uncropped Western Blot images of Fig. 8C (left). protein expression levels of PNPLA8, phospho-Akt (ser473), Akt, phospho-Gsk3β (ser9), phospho-Erk1/2 (Thr202/Tyr204), Erk1/2 and GAPDH in SUM159PT cell lines treated with PNPLA8 siRNA or scrambled siRNA combined with the treatment of PGE2 (1 μM) or 20-HETE (10 nM) for 48 hours. GAPDH was used as loading control.

Figure S12

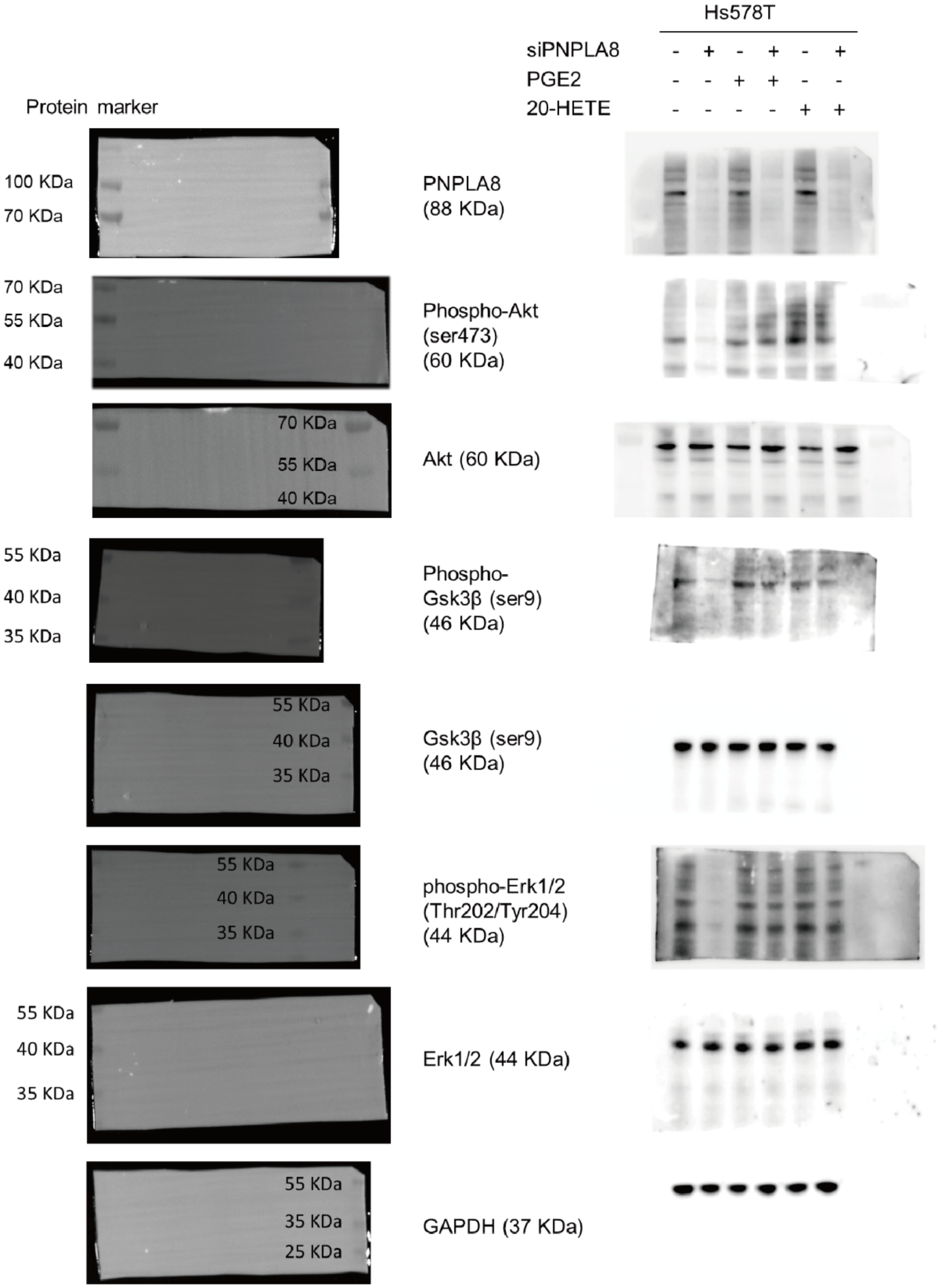

Figure S12. Full uncropped Western Blot images of Fig. 8C (right). Protein expression levels of PNPLA8, phospho-Akt (ser473), Akt, phospho-Gsk3β (ser9), phospho-Erk1/2 (Thr202/Tyr204), Erk1/2 and GAPDH in Hs578T cell lines treated with PNPLA8 siRNA or scrambled siRNA combined with the treatment of PGE2 (1 μM) or 20-HETE (10 nM) for 48 hours. GAPDH was used as loading control.
